# Barriers to, and Facilitators of, Exercising in Fitness Centres among Adults with and without Physical Disabilities: A Scoping Review

**DOI:** 10.3390/ijerph18147341

**Published:** 2021-07-09

**Authors:** Helene Nikolajsen, Louise Fleng Sandal, Carsten Bogh Juhl, Jens Troelsen, Birgit Juul-Kristensen

**Affiliations:** 1 Research Unit for Musculoskeletal Function and Physiotherapy, Department of Sports Science and Clinical Biomechanics, University of Southern Denmark, 5230 Odense, Denmark; cjuhl@health.sdu.dk (C.B.J.); bjuul-kristensen@health.sdu.dk (B.J.-K.); 2 Department of Physiotherapy, Institute of Health Studies, University College South Denmark, 6705 Esbjerg Ø, Denmark; 3 Research Unit for Physical Activity and Health in Work Life, Department of Sports Science and Clinical Biomechanics, University of Southern Denmark, 5230 Odense, Denmark; lsandal@health.sdu.dk; 4 Department of Physiotherapy and Occupational Therapy, Copenhagen University Hospital, Herlev and Gentofte, 2900 Hellerup, Denmark; 5 Research Unit for Active Living, Department of Sports Science and Clinical Biomechanics, University of Southern Denmark, 5230 Odense, Denmark; JTroelsen@health.sdu.dk

**Keywords:** fitness centre, gym, disabilities, contextual factors, accessibility, personal factors, fitness instructors, social connections, scoping review

## Abstract

Fitness centres are an obvious arena for performing physical activity for the general population but representation of adults with physical disabilities (AwPD) is lacking. To increase possibilities for AwPD to exercise in fitness centres together with adults without physical disabilities (AwoPD), the aim of this study was to identify, synthesise, and compare barriers to, and facilitators of, exercising in fitness centres for each group. A scoping review was conducted and data extraction of the barriers and facilitators was performed independently by two researchers on six categories of contextual factors based on the framework of Di Blasi: (1) The fitness centre setting; (2) The fitness centre user characteristics; (3) The fitness instructor/staff characteristics; (4) The fitness centre user–instructor/management relationship; and (5) The fitness/exercise characteristics. An extra category, (6) Other relationships, was added. The PRISMA Extension for Scoping Reviews was used for reporting. Of the 102 included papers, only 26 (25%) of the papers were on AwPD, which focused mainly on physical barriers (category 1: inaccessible settings). In contrast, the remaining 76 papers involving AwoPD focused primarily on facilitators (category 2: motivational factors and exercising effects). In categories 3–6, the two groups had similar results, as both groups preferred skilled instructors, a welcoming and comfortable fitness centre environment, an ability to exercise at their preferred type and level, and good social connections. Since most data were based on AwoPD, more studies on actual experiences from AwPD are needed, to reveal the facilitators/motivational factors for fitness centre use.

## 1. Background

Globally, 27% of the adult population does not meet the general recommendations for engaging in physical activity [1], which poses a threat to public health, and constitutes a significant risk for developing non-communicable diseases [2]. However, a Danish survey revealed that 71% of those reporting to be physically inactive stated that they would like to be more physically active [3], which indicates the potential for increasing physical activity levels among the inactive population. 

Physical activity can contribute to the prevention of a broad spectrum of diseases [4,5], and the World Health Organisation (WHO) recommends adults perform at least 150 min of moderately intense physical activity every week, or a minimum of 75 min of vigorously intense activity each week distributed across three weekdays, or an equivalent combination of moderate and vigorous activity [6,7]. This recommendation holds true for both adults with and without physical disabilities [8]. Fulfilling these recommendations would seem more beneficial for people with disabilities, as they are less physically active and, as a consequence, experience more diseases at an earlier age [9,10]. However, this group experiences a long list of both socially and environmental barriers [11,12], which makes it even harder to fulfil the WHO recommendations.

Physical activity is often performed through leisure-time activities in high-income countries [13] and exercising in fitness centres may, therefore, be a means of increasing physical activity in the general population. Fitness centres have gained in popularity since their inception in the USA in the early 1970s [14], and today are considered the world’s biggest ‘sport’ [15]. The USA is the leading market with a penetration rate of 20.8% in 2018 [16]. In Europe, membership rates of commercial fitness centres have grown 3.8% from 2018 to 2019, resulting in 9.7% of the people above 15 years being members, and with potential for further growth [17]. Their popularity may be due to the variety of exercising opportunities that can be adjusted to the individual user according to their preferences, e.g., flexible hours with structured or unstructured activities performed in groups or individually, and a variety of exercising possibilities that suit the beginner, the advanced, and the professional user [14,18,19]. This aligns very well with the preferred choices of physical activity by people with disabilities, as they generally prefer activities that they can take part in alone, with low demand for organisation and rules [20].

Generally, research within fitness centre settings has either focused on cultural or sociological aspects [21], or on the more extreme aspects of fitness centre environments, such as bodybuilding [22], orthorexia [23], performance-enhancing factors, such as doping [24], or nutrition/dietary supplements [25]. Research on the largest or most frequent group of people training in fitness centres is needed, especially regarding daily experience of fitness training as a way of increasing physical activity among the general population. Knowledge about people with disabilities and their experience (positive and negative) with fitness centres is also sparse, and it is anticipated that one of the reasons is that fitness centre accessibility for this group is limited [26]. From a societal point of view, this lack of knowledge is problematic because people with disabilities (physical as well as mental) constitute a growing group of more than a billion people or about 15% of the world’s population, and with the prolonged life expectancy of this group, continued growth is expected [27]. 

Frequently cited reasons for not being as physically active as one would like are lack of time, energy, and motivation [28]. For people with physical disabilities, barriers such as negative attitudes from other people and inadequate policies and standards, besides the inaccessible surroundings, have been reported [27]. To increase the possibility of participation in exercising in fitness centres for both AwoPD and AwPD, more information is needed on the barriers and facilitators in order to increase the levels of physical activity and thereby reduce the risk of lifestyle diseases.

There is a knowledge gap in the scientific systematic compilation of the barriers to and facilitators (not only the physical ones) of performing physical activity in fitness centres for AwPD. Further, since AwoPD is the dominant group in regular fitness centres, it is also important to know the experiences associated with these barriers and facilitators for AwoPD, so that AwPD and AwoPD can perform physical activity together in the fitness centres. Moreover, the WHO calls for safe, accessible, affordable, and appropriate spaces to be physically active in the Global Action Plan on Physical Activity 2018–2030 [29], and stresses special attention be paid to vulnerable groups; i.e., people with disabilities and chronic diseases.

Therefore, the aim of this study was to identify, synthesise, and compare the barriers to, and facilitators of, exercising in fitness centres among groups of adults with physical disabilities (AwPD) and adults without physical disabilities (AwoPD). 

## 2. Methods

### 2.1. Methodological Design

To provide an overview of the barriers and facilitators associated with exercising in fitness centres among adults with and without physical disabilities, a scoping review was conducted. Scoping reviews are fruitful when a body of literature has not previously been comprehensively reviewed, is heterogeneous in nature [30], or implies different indications [31]. 

A five-step protocol was used for conducting the scoping review, as previously recommended [30,32,33,34], based on the framework of Arksey and O’Malley [35] and Levac [36]. An a priori protocol for this scoping review was made publicly available online, at the European Open Access Science Repository Zenodo.com on 5 September 2018 (doi:10.5281/zenodo.1409587) (accessed on 02 October 2018) [37]. Furthermore, the PRISMA Extension for Scoping Reviews (PRISMA-ScR) [38] was used as a guideline for reporting. 

### 2.2. Step 1—Identifying the Research Question

The research question to be explored was: Which contextual factors are perceived as barriers to, and facilitators of, fitness centre participation amongst adults with or without physical disabilities? Contextual factors were grouped a priori into categories based on the Di Blasi framework [39], previously used to describe context effects in practitioner–patient interactions. Di Blasi and colleagues proposed five categories to describe the context surrounding any health care situation that may influence the effect of interactions. This includes the practitioner–patient interaction in relation to the practitioner’s acting, talking, and behaving, which may positively or negatively influence the effect of the treatment. Consequently, the framework is used as a model for categorising the barriers and facilitators. In this review, adjusting the category labels and adding an extra category (‘Other relationships’) were performed to target the fitness centre setting. Therefore, the six categories were (1) The fitness centre setting; (2) The fitness centre user characteristics; (3) The fitness instructor/staff characteristics; (4) The fitness centre user–instructor/management relationship; (5) The fitness/exercise characteristics; and (6) Other relationships (Table 1).

### 2.3. Step 2—Identifying Relevant Studies

To capture the core elements of the research question, we used the Population, Concept and Context (PCC) mnemonic, as previously recommended [33], to determine the inclusion criteria. The included ‘Population’ comprised adults above 18 years of age (a common age restriction in fitness centres), with or without physical disabilities. The ‘Concept’ incorporated the variety of contextual factors encouraging or hindering participation (e.g., transportation, usability, accessibility, motivation, and affordability), and the ‘Context’ was limited to indoor fitness centre/gym/health club settings where people exercise voluntarily in their leisure time. The exclusion criteria were people with cognitive disorders/mental illness (depression, psychiatric diagnosis, etc.), participation in prescribed (non-voluntary) exercise types that were done as part of rehabilitation in the healthcare sector, and exercising in worksite fitness centres where the public did not have access. Furthermore, because the primary focus was on the most common fitness centre user, rather than niche groups, a few records that focused on the experience of LGBTIQ+ or cultural or religious populations were excluded. Moreover, records were also excluded if the main focus of the record was on performance and intake of drugs or nutrition/dietary supplements, investigating different aspects of extreme behaviour, such as orthorexia, bodybuilding, and weightlifting, or focusing solely on body image, weight loss/obesity, hygiene and bacteria levels, or defibrillators and heart attacks in fitness centres. We included records published in English, Danish, Norwegian, and Swedish.

All types of scientific records involving both quantitative and qualitative designs were included for original studies and reviews. ‘Grey literature’, such as theses, conference proceedings, research reports, government reports, policy statements, fact sheets, and articles from newspapers and magazines, etc., were included, as proposed in the PRISMA-ScR [38]. Furthermore, no restriction on publication date was applied.

#### Search Strategy 

We utilised a three-step protocol, as previously mentioned [33]. Firstly, we performed a cursory search using google.com, including Google Scholar, duckduckgo.com, and the electronic databases Medline and Cinahl, to identify the relevant search terms. 

Secondly, guided by a medical research librarian, a block strategy using Boolean operators was constructed (see Supplementary S1). Index terms were adjusted and tailored for each of the six databases (Medline (via PubMed), Scopus (via Elsevier), Cinahl and SPORTDiscus (via EBSCO), and PsycInfo and Embase (via Ovid)). The initial search was performed on 15 October 2018, with an update undertaken on 19 May 2020. 

Thirdly, we conducted a systematic search for the ‘grey literature’ using relevant parts of the Canadian Agency for Drugs and Technologies in Health (CADTH) guidelines [41], as proposed in the PRISMA-ScR [38]. Librarians with field expertise at The Danish Disability Sport Information Centre and Marselisborg Centre, Aarhus University Hospital, were contacted for references and advice for further search strategies. Finally, google.com, including Google Scholar and the University Library database, ‘Summon’, at the University of Southern Denmark, were used to search for additional relevant literature. Further citation searching and searches of key authors were performed in all parts of the ‘grey literature’ search.

### 2.4. Step 3—Study Selection

All records were imported from Endnote X9 to Covidence (https://www.covidence.org, accessed on 21 May 2021), an online systematic review platform, and checked for duplicates.

Title and abstract screening of all records were performed independently by two people (a physiotherapy student (N.K.L.) and the first author (H.N.)), to exclude all obvious irrelevant records (e.g., animal trials). Subsequently, a title and abstract screening for eligibility were performed. Reviewer 1 (H.N.) screened all records, while Reviewer 2 (L.F.S.) and Reviewer 3 (B.J.K.) screened half of the records each. All references were screened independently by the reviewers and consensus was achieved, with any conflicts resolved by discussion.

**Figure 1:**
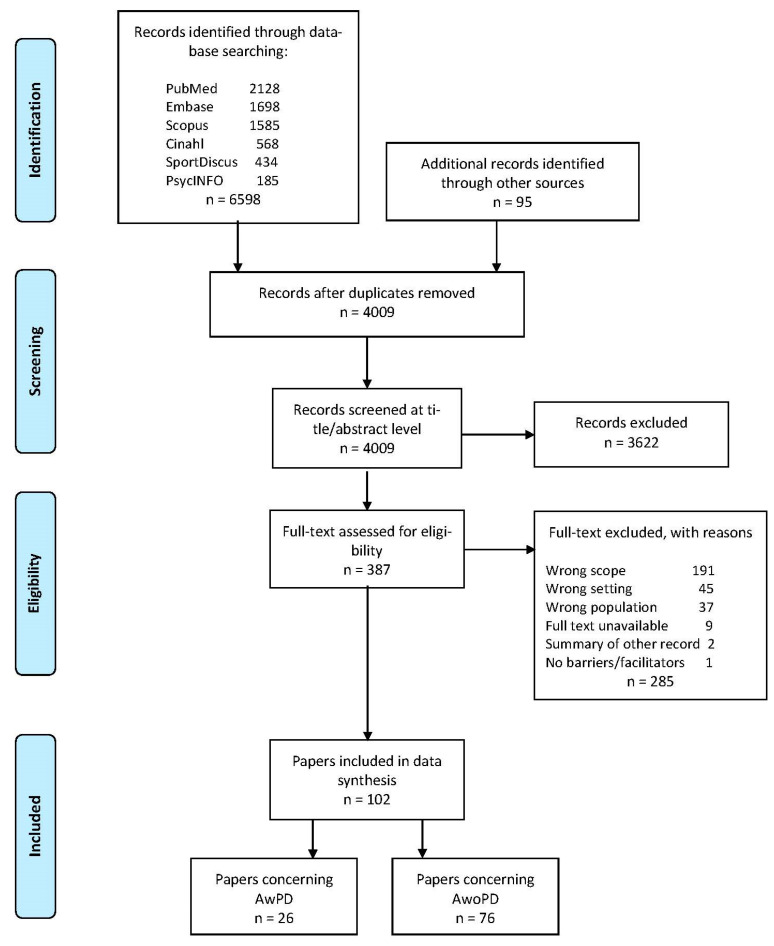
Study selection process illustrated in a PRISMA flowchart. AwPD = adults with physical disabilities; AwoPD = adults without physical disabilities.

Thereafter, a full-text screening was performed independently by two reviewers using the same method as described above. During the screening process, two consensus meetings reinforced a common understanding of the inclusion and exclusion criteria. A flowchart of the process is presented in the Results section (Figure 1).

### 2.5. Step 4—Charting the Data (Data Extraction Process)

Data extraction was performed using a customised Excel data extraction sheet (see Supplementary S2), containing the following categories: General characteristics—author(s), year of publication, origin (where the study was conducted), type of publication, aim/purpose, and methodology/methods; Population—characteristics and numbers; grouping of Concept (the contextual factors) into the six categories (Table 1) [39]; and Context—the type of indoor fitness centre. Data extraction was performed independently by two reviewers and conflicts were resolved by discussion.

Barriers and facilitators were defined as everything that could hinder or enable exercising in fitness centres, and if not directly described in the text, a common-sense approach was used for categorising a factor as either a barrier or a facilitator. We established a standard set of rules before extracting data from the included papers, which consisted of a variety of study types, to determine when a factor could be labelled as a barrier or a facilitator:

Quantitative data:

- Descriptive studies (e.g., questionnaires)—if more than 50% of the respondents stated the factor as a barrier or a facilitator;
- Regression/correlation analysis—a significant result according to the definition in the paper;
- Factor analysis—a significant result according to the definition in the paper.
- Qualitative data:
- Papers with a results section—barriers or facilitators described in the results or conclusion sections;
- ‘Grey literature’ without a results section—if barriers or facilitators were described in the text.

Under each of the six categories (Table 1), barriers and facilitators were grouped with headlines and sub-points and ordered in a pragmatic chronology, rather than indicating importance or data saturation. Results from the two groups, AwPD and AwoPD, were kept separately. 

### 2.6. Step 5 Collating, Summarising, and Reporting the Results

The Results section consists of three parts: Firstly, a numerical summary of the number of included records (Figure 1), to establish an overview of the general characteristics, such as publication year, origin, type, and population included (Table 2, and Supplementary Table S3). Secondly, a descriptive summary of the barriers and facilitators grouped in categories is presented, and reported separately for the two groups, AwPD (Table 3 and Table 4) and AwoPD (Table 5 and Table 6). Thirdly, a comparative analysis of the similarities and differences concerning the barriers and facilitators for the groups is presented (Table 7). 

## 3. Results

### 3.1. Numerical Summary

We identified 6598 records through the six scientific databases, and 95 records through other sources in our search for unpublished and ‘grey literature’. After removal of duplicates, 4009 unique records were identified (Figure 1). 

Of those, a total of 102 papers were included in the scoping review (Supplementary Table S3, alphabetic list by first author). All papers were published between 1995 and 2020 and were from five continents (North America = 58; Europe = 36; Oceania = 5; Asia = 2; and South America = 1). Of the 102 papers, about 75% were scientific papers of original studies using quantitative, qualitative, or mixed methods. The remaining 25% were categorised as ‘grey literature’ and consisted of a broad spectrum of reports and guidelines, and articles from newspapers or magazines (Table 2). 

**Table 2:** Overview of the 102 included papers: 26 papers [11,26,42,43,44,45,46,47,48,49,50,51,52,53,54,55,56,57,58,59,60,61,62,63,64,65] on adults with physical disabilities (AwPD) and the remaining 76 papers [66,67,68,69,70,71,72,73,74,75,76,77,78,79,80,81,82,83,84,85,86,87,88,89,90,91,92,93,94,95,96,97,98,99,100,101,102,103,104,105,106,107,108,109,110,111,112,113,114,115,116,117,118,119,120,121,122,123,124,125,126,127,128,129,130,131,132,133,134,135,136,137,138,139,140,141] on adults without physical disabilities (AwoPD).

	Type of Paper	AwPD Reference Number	n	%	AwoPD Reference Number	n	%
Scientific	Quantitative studies	[44,45,46,47,50,55,62]	7	27	[69,74,75,76,77,80,81,82,83,85,86,87,90,91,92,93,94,98,100,103,108,110,118,119,120,122,123,125,133,134,135,136,137,139,140]	36	47
	Qualitative studies	[11,51,58,59,60]	5	19	[66,70,79,84,88,99,102,105,106,107,113,117,121,126,130]	15	20
	Mixed method studies	[43]	1	4	[71,72,114,115,124,132,138]	7	9
	Systematic reviews	[26]	1	4			
	Reviews/opinion papers	[61]	1	4			
	Theses				[89,116]	2	3
Grey	Conference papers	[48]	1	4	[68]	1	1
	Conference poster				[101]	1	1
	Guidelines	[49,52,53,56,64,65]	6	23			
	Reports				[67,95,96,111,112]	5	7
	Magazine articles	[54,57,63]	3	11	[78,97,104,109,131]	5	7
	Newspaper articles	[42]	1	4	[73,127,128,129]	4	5
	In total		26	100		76	100

### 3.2. Descriptive Summary

#### 3.2.1. Adults with Physical Disabilities (AwPD)—Barriers and Facilitators

Of the 102 included papers, only 26 [11,26,42,43,44,45,46,47,48,49,50,51,52,53,54,55,56,57,58,59,60,61,62,63,64,65] included AwPD. Of these 26 papers, almost 60% could be categorised as scientific literature, and the remaining grey literature were conference papers, guidelines, magazines, and newspaper articles. Only six papers included experiences from AwPD themselves [11,42,51,58,59,60] (of which one was a short newspaper article, and three were from the same author group with an overlapping study population). The included group of AwPD had a very heterogenous level of physical impairment, which was poorly described. Diagnoses included cerebral palsy, spinal cord injury, post-polio syndrome, Parkinson’s disease, injuries from accidents, fibromyalgia, and back problems. The remaining 18 papers dealt with outside perspectives (researchers, disability associations, fitness managers, etc.). Those of fitness managers included options intended for AwPD, whereas the other outside perspectives were guidelines on how to make fitness facilities accessible and usable.

For AwPD in general, the focus was mainly on the barriers that explained why AwPD rarely use fitness centres, most of which were due to accessibility issues and non-adjustable equipment, corresponding to 18 out of 26 papers (Table 3, first column). Negative interactions with other people, both instructors/staff and other users, were also reported as barriers to fitness centre participation. Fourteen different subgroups of barriers (Table 3) and 12 different subgroups of facilitators (Table 4) were identified. Consequently, facilitators of fitness centre participation for AwPD were lacking, e.g., the motivational factors for exercise adherence and advantages/effects of physical exercise. 

**Table 3:** Barriers to exercising for adults with physical disabilities (AwPD) distributed across the six modified context factor categories. Numbers in parentheses in the coloured cells refer to the total number of different papers (references in the square brackets) informing each of the six categories.

1. The Fitness Centre Setting (18 Papers)	2. The Fitness Centre User Characteristics (6 Papers)	3. The Fitness Instructor’s/Staff Characteristics (7 Papers)	4. The Fitness Centre User—Instructor/Management Relationship (9 Papers)	5. The Fitness/Exercise Characteristics (5 Papers)	6. Other Relationships (7 Papers)
**Poor transportation options** [11,26,42,45,50,52,55] - Poor public transportation - Parking lots; too few, wrong dimensions, or lack of curve cuts	**Lack of knowledge about accessible and available facilities** [11] - Potential users do not know about the inclusive facilities	**Lack of skilled instructors** [11,26,43,45,50,52,53] - Lacking knowledge of disabilities, accessibility issues, wheelchair transfer, exercise/therapeutic exercise and available programs and services	**Management not being actively inclusive** [11,43,52,53,56,57,62] - Conscious or unconscious discrimination - Lack of policies for service animals - Focus on youth and physical prowess - Prioritising of profit over accessibility	**Lack of tailored classes/ adaptive programs** [11,26,42,46,52] - People with different disabilities not given different types of exercises - Group classes are not accessible and/or usable - Concerns about needing/requesting assistance	**Stigma from non-disabled members leading to direct psycho-emotional disablism** [51,52,53,59,60] - Negative attitudes from other members - Disability is an unknown phenomenon to many non-disabled people
**Poor accessibility to the fitness centre and bathrooms/locker rooms** [11,26,44,45,47,50,51,52,53,55,57,58,59,61,62,63] - Stairs/no elevators - Lack of floorspace and obstructed pathways - Doors; poor grasp function, too heavy or too narrow - Lack of benches and additional seating when resting, getting dressed or showering - Toilets, grasp bars, soap and toilet paper dispensers, mirrors etc. placed out of reach	**High costs** [11,42] - Transportation and memberships are perceived as high cost - Charging additional membership costs for attending personal assistants		**Negative attitudes resulting in direct psycho-emotional disablism** [11,43,52,53,59,60] - Staff and managers tend to view accessibility as a ‘necessary evil’ or as unimportant - Not an accepting or inviting attitude		**Negotiations about body ideals, rights and power** [58] - The stereotypical ideal body of a ‘normal’ and fit body being predominant
**Unsuitable fitness equipment** [11,26,45,47,48,50,52,55,57,59,63] - Seats are too small to transfer to and are not movable - Lack of specialised, adaptive and accessible equipment, e.g., cardiovascular and upper body only	**Negative feelings about fitness** [11,51,58,59,60] - Fear of the unknown and anticipation of the fitness centre as an exclusive space - Feeling unwelcomed, under-represented or misunderstood when being at the fitness centre - Feeling othered, embarrassed or ashamed of their body and not fitting into the ‘normal’ body ideal		**Lack of knowledge leading to unprofessional assistance** [56,60] - Not knowing how to assist people with physical disabilities - Different understanding of pain, as in warning or ‘no pain no gain’		**Lack of support from friends and families** [11] - Resulting in lack of motivation and participation

**Table 4:** Facilitators of exercising amongst adults with physical disabilities (AwPD) distributed across the six modified context factor categories. Numbers in parentheses in the coloured cells refer to the total number of different papers (references in the square brackets) informing each of the six categories.

1. The Fitness Centre Setting (14 Papers)	2. The Fitness Centre User Characteristics (2 Papers)	3. The Fitness Instructor’s/Staff Characteristics (9 Papers)	4. The Fitness Centre User—Instructor/Management Relationship (8 Papers)	5. The Fitness/Exercise Characteristics (7 Papers)	6. Other Relationships (5 Papers)
**Universal design/good accessibility** [11,44,47,49,53,54,56,57,65] - Removal of physical barriers inside and outside - Wheelchair-friendly surroundings - Automatic doors or power door openers - Extra floor space - Family locker rooms	**Benefits from exercising** [60] - Physical improvements, e.g., improved function, reduced pain, improved fitness, enhance independence - A break that gives an energy boost	**Specially trained staff** [11,43,44,50,55,56,58,64] - Staff who can adapt existing fitness classes to people with disabilities, know how to exercise safely and effectively and know when to stop - Disabled fitness instructors having better skills to adapt equipment/exercises - Managers supporting the education of their staff and hiring those with these adaptive skills	**Correct guidance and assistance from instructors** [56] - Listening to instructions from the individual which provides the best way to assist them - Offering assistance, but waiting until the offer is accepted before helping - Treating the wheelchair as an extension of their body	**Tailored exercise programs to people with physical disabilities** [47,53,55,56,57,63,64] - Programs and classes for all fitness levels - Different classes, e.g., introductory classes, chronic illness classes or aerobics while seated - Offering assistance with accessible and adaptable equipment - Evidence-based and activity-based interventions	**The fitness centre as a social arena** [51,58,59,60,63] - Making new friends and meeting peers - Disabled peers who act as role models and friends who encourage and support - Teaming up and having fun with friends - Acting on an even playing field with non-disabled people
**Specialised fitness equipment** [11,44,45,47,52,56,57,63,64] - Offering a wide variety of both strength and cardio exercises - Equipment easy to enter and exit or with swing-away seats so no transferring is needed - Adaptive equipment for gripping, e.g., gloves, hooks, mitts, cuffs - Supportive aids for extra balance, e.g., long Velcro straps or belts, pedal straps, toe clips, weight belts, wedges - Low weights (from ½ kg) and small increments in weight equipment (from 2.5 kg) - Raised ‘treatment table’ or elevated mats for floor exercises	**Positive experiences related to fitness** [59,60] - Feeling empowered and integrated in the gym - Psychological respite, from stress associated with having a disability	**Respectful communication** [11,49,56] - Being friendly and interacting with people with physical disabilities as with any other member - Allowing extra time and having an open communication about abilities and limitations	**Inclusive and tolerant environment** [51,56,58,60] - Disabled fitness instructors acting as role models - Disabled instructors and members who challenge the stereotypical body ideal in fitness settings, and focus on health and personal progress - Marketing materials showing people with physical disabilities/older adults - Comfortable, friendly environment with a sense of community		
**Use of checklists to improve accessibility** [49,56,61] - Use of checklist and guidelines like AIMFREE (Accessibility Instruments Measuring Fitness and Recreation Environments), ADA checklist or Fitness Facilities: An Abbreviated Accessibility Survey			**Membership/low costs** [11,44,50,55,56] - Personal assistants who accompany the clients at the facility free of charge - Offering free trials visits - Sliding fee scale or scholarships		

#### 3.2.2. Adults without Physical Disabilities (AwoPD)—Barriers and Facilitators 

Of the 76 papers [66,67,68,69,70,71,72,73,74,75,76,77,78,79,80,81,82,83,84,85,86,87,88,89,90,91,92,93,94,95,96,97,98,99,100,101,102,103,104,105,106,107,108,109,110,111,112,113,114,115,116,117,118,119,120,121,122,123,124,125,126,127,128,129,130,131,132,133,134,135,136,137,138,139,140,141] identified on AwoPD, almost 80% were categorised as scientific literature (Table 2). The group of AwoPD seemed more homogenous and was mostly sub-grouped based on age, gender, and membership status, such as being new users or long-time/regular users. Twelve different subgroups of barriers (Table 5) and 13 subgroups of facilitators (Table 6) were identified. For AwoPD, the papers mainly focused on facilitators, corresponding to 43 of the 76 papers (Table 6, column two), and the primary focus was on personal motivation, exercise effects, and exercise adherence. 

**Table 5:** Barriers to exercising for adults without physical disabilities (AwoPD) distributed across the six modified context factor categories. Numbers in parentheses in the coloured cells refer to the total number of different papers (references in the square brackets) informing each of the six categories.

1. The Fitness Centre Setting (8 Papers)	2. The Fitness Centre User Characteristics (22 Papers)	3. The Fitness Instructor’s/Staff Characteristics (4 Papers)	4. The Fitness Centre User—Instructor/Management Relationship (6 Papers)	5. The Fitness/Exercise Characteristics (2 Papers)	6. Other Relationships (8 Papers)
**Long transportation time/distance to fitness centre** [69,71,126] - Long distance to travel and crowded parking lots	**Dislike of the fitness culture** [70,97,99,113,122,123,131] - Discouragement due to the stressful and competitive atmosphere of gyms - Dislike the ‘ideal’ body attitudes of skinny woman in skimpy spandex and men with rock hard abs - Lacking in confidence or feeling embarrassed about their body or clothes	**Lack of professional guidance** [70,106,107,122] - Lack of practical skills or solid educational background, resulting in faulty guidance, pain or injuries - Lack of social skills	**Negative staff attitudes** [79,97,107,120,122] - Over-ambitions instructors - Judgemental, unethical, unprofessional and intimidating staff - Lack of respect, attention and punctuality from the staff	**Uninteresting/boring exercises** [115,117] - Use of the gym equipment seen as boring and not appealing/enjoyable	**Lack of social connections** [113,115] - Loss of spouse or their workout partner makes older people stop exercising in the fitness centre - Absence of social connections negatively affects motivation
**Unattractive fitness facilities** [71,100,113,122,123,131] - Noise levels/loud music - Unpleasant odours, poor hygiene/cleanliness - Limited equipment or inadequate equipment for obese/larger size people - Poor safety of lockers	**Lack of knowledge** [70,71,84,90,104,113,123] - Lack of basic understanding of benefits of exercising - Lack of knowledge about how to adjust exercise to suit health problems, medical conditions or pregnancy		**Body ideals and physical performance** [97,115,122] - Super skinny and fit fitness instructors who scare the not-so-fit users - Disbelief or demoralising comments related to poor physical performance - Stigmatising slogans and images in the fitness centre		**Lack of support from health authorities** [113] - Lack of public education campaigns about fitness for older adults - Lack of health practitioner advice
	**Individual priorities** [70,71,73,80,85,90,95,96,99,105,111,112,113,116,117,123,126] - Lack of time, energy or being too busy with other things - Not interested or motivated - Poor weather or seasonal conditions or holidays - Not a member of a fitness club /short membership time or few entrances - Membership fees are too high or the existence of returned receipts - Lack of a workout body - Having pain or injury				**Not fitting in** [71,78,113,116,122,128,131] - Unwelcome environment - Blame and stigmatisation because of body appearance or age - Not knowing the gym etiquette, newbies vs. gym rats - Social anxiety/doubt about own capabilities

**Table 6:** Facilitators of exercising for adults without physical disabilities (AwoPD) distributed across the six modified context factor categories. Numbers in parentheses in the coloured cells refer to the total number of different papers (references in the square brackets) informing each of the six categories.

1. The Fitness Centre Setting (18 Papers)	2. The Fitness Centre User Characteristics (43 Papers)	3. The Fitness Instructor’s/Staff Characteristics (15 Papers)	4. The Fitness Centre User—Instructor/Management Relationship (13 Papers)	5. The Fitness/Exercise Characteristics (14 Papers)	6. Other Relationships (21 Papers)
**Easy access** [67,70,71,87,91,102,110,114,117,132] - Located near home or work - Transport time a maximum of 15–30 min	**Health and body appearance** [66,67,68,70,73,75,79,84,86,90,95,96,99,102,105,110,111,112,113,114,115,117,121,125,126,127,128,129,130,132,133] - Exercising because of the positive impact on the body and physical well-being - Desire to lose or control body weight - Wanting to maintain/improve physical fitness, e.g., get stronger or enhance endurance maybe for work or other sports - Gaining an attractive, good-looking and fit body - Preventing or reducing pain and other discomforts or managing chronic health conditions - Older people also having focus on fighting some of the negative effects of ageing, e.g., being able to perform daily tasks and other activities and stay independent. - Visiting the gym perceived as a health investment in the future	**The ideal instructor** [70,71,72,73,74,100,102,103,106,107,110,117,124,131,138] - Appropriate level of knowledge/skills, e.g., college degree or other good certifications - Good social skills, being friendly, kind and helpful - Qualities of being engaged, dedicated approachable, visible, empathic, motivating and making exercising fun - Good physical appearance which is important in for-profit settings	**Comfortable atmosphere** [66,76,94,115,124] - A comfortable and welcoming feeling for new members - Diversity in instructors/staff which is important in non-profit settings and is a way of promoting inclusion - Members becoming instructors which helps to influence the fitness centre and gain co-responsibility	Fitness classes [70,100,101,110,117,126,127,129,131] - Wide variety of classes to fit personal preferences and fitness levels - Specially tailored classes for, e.g., seniors, family workout or parent-and-baby fitness classes - Use of structured daily programs which can enhance retention	**Social connections** [66,70,71,81,84,88,97,102,105,113,114,115,117,121,124,126,132,134,138,139,140] - A place to meet peers and new and old friends - Group activities which are good for social interaction - Other people act as role models - Motivation to exercise in groups, makes it interesting and fun and provides social support - ‘Feeling of belonging or being a part of a community - Group perceptions and satisfaction which predict attendance
**Pleasant fitness environment** [88,91,98,109,110,124,127,129,131,132,141] - Well-maintained locker rooms and showers - Good variety, up-to-date equipment for the right functional level (from physically dependent to elite level) - Not too crowded, which makes easy access to the equipment - Women-only areas - Room for children, childcare, classes for children or families - Positive visual images of people of all sizes enjoying physical activity - No mirrors or areas with limited number of mirrors	**Positive mind and feelings** [68,70,73,79,84,86,89,90,95,96,99,102,110,111,112,114,115,117,118,126,130,132] - Enhanced mental well-being and feeling good, e.g., relaxation, more energy, better mood and sleep - Self-motivating, where exercising is fun and enjoyable - Feeling of being healthier and happier, builds confidence and the feeling of being empowered - Being disciplined and in control, evokes feelings of e.g., pride, self-confidence, satisfaction, capability and autonomy - Combating negative feelings e.g., stress, depression, frustration, anxiety or anger - Self-identification as an active person and the feeling of having bettered themselves and moved ‘up’ as well as ‘out’ of their own social class		**Soft values** [74,94,97,113,115,124,126,138] - The instructor/staff who acts professionally; makes the participant feel welcome, important and not judged regardless of fitness level, size etc. - Motivating, supporting, encouraging and ensuring appropriate levels of assistance - Setting small goals to build up confidence and gain trust with the unfamiliar/new fitness user - Keeping the workouts fun and consistent to increase the likelihood of habit formation	**Individual focus/goal** [76,104,108,110,116,138] - Individually tailored programs developed by skilled personal trainers, e.g., based on pre-exercise evaluation - Personal goals are supported by individual programs and tracking of progress - Use of coaching sessions or motivational interviewing for further progress - Use of individualised small-group workouts	
	**Feeling comfortable in the fitness centre** [67,70,71,72,92,93,94,99,110,113,114,115,119,121,131,132,137] - Past behaviour—e.g., good childhood/youth experience with sport/exercise - Establishing fitness centre exercising as a habit, at a convenient time and location - Identifying as a member, as a part of self-identity - Social connections, meeting new people or having friends or family to train with - Feeling welcomed, valued and comfortable in the centre, with a caring, positive and supportive climate - Exercising which leads to satisfaction, autonomy, competence, enjoyment etc. - Inclusion, the feeling of fitting in with respect to age, looks and room for making mistakes - Having the skills to practically and technically operate the equipment - Exercising at one’s own pace		**Membership** [66,76,83,135,136] - Low membership fees and, e.g., seniors’ discount - Possibility of short enrolment, e.g., only one month - Commitment lotteries, e.g., exercise x times a month and having the chance of winning a month’s free membership - Loyalty programmes, e.g., earning air miles bonus points		
	**Low costs** [70,71,77,91,113,114] - Inexpensive or free exercise programs (e.g., paid by health care or insurance, or under $100 per month) - One month’s free membership is an effective reinforcer for attendance at the fitness facility (exercise 12 times in a month to earn it)				

### 3.3. Comparative Analysis 

The amount and type of papers (scientific/grey) differed between the two groups, where the quantity and quality of research were more comprehensive in AwoPD compared with AwPD (Table 2). Further, for AwoPD, the study designs were relatively homogeneous, including many quantitative designs differing on, e.g., gender, age, and exercise experience. This was in contrast to the AwPD, where the papers were heterogeneous with respect to type (many grey) and diagnoses. The participants had different levels of physical ability but were mainly described as one collective group.

Furthermore, the main focus differed. For AwPD, the main focus was on barriers, while for AwoPD, it was on facilitators (Table 7).

**Table 7:** Overview of the barriers and facilitators for the two groups (Table 3, Table 4, Table 5 and Table 6), related to the six context factor categories. Numbers in parentheses refer to the total number of different papers informing each of the subgroups.

	Adults with Physical Disabilities (AwPD)		Adults without Physical Disabilities (AwPD)	
Context Factor Categories	Barriers (Table 3)	Facilitators (Table 4)	Barriers (Table 5)	Facilitators (Table 6)
**1. The Fitness Centre Setting**	Poor transportation options (7) Poor accessibility to the fitness centre and bathrooms/locker rooms (16) Unsuitable fitness equipment (11)	Universal design/good accessibility (9) Specialised fitness equipment (9) Use of checklists to improve accessibility (3)	Long transportation time/distance to fitness centre (3) Unattractive fitness facilities (6)	Easy access (10) Pleasant fitness environment (11)
**2. The Fitness Centre User Characteristics**	Lack of knowledge about accessible and available facilities (1) High costs (2) Negative feelings about fitness (5)	Benefits from exercising (1) Positive experiences related to fitness (2)	Dislike of the fitness culture (7) Lack of knowledge (7) Individual priorities (17)	Health and body appearance (31) Positive mind and feelings (22) Feeling comfortable in the fitness centre (17) Low costs (6)
**3. The Fitness Instructor’s/Staff Characteristics**	Lack of skilled instructors (7)	Specially trained staff (8) Respectful communication (3)	Lack of professional guidance (4)	The ideal instructor (15)
**4. The Fitness Centre User —Instructor/Management Relationship**	Management not being actively inclusive (7) Negative attitudes resulting in direct psycho-emotional disablism (6) Unprofessional assistance (2)	Correct guidance and assistance from instructors (1) Inclusive and tolerant environment (4) Membership/low costs (5)	Negative staff attitudes (5) Body ideals and physical performance (3)	Comfortable atmosphere (5) Soft values (8) Membership (5)
**5. The Fitness/Exercise Characteristics**	Lack of tailored classes/adaptive programs (5)	Tailored exercise programs to people with physical disability (7)	Uninteresting/boring exercises (2)	Fitness classes (9) Individual focus/goal (6)
**6. Other Relationships**	Stigma from non-disabled members leading to direct psycho-emotional disablism (5) Negotiations about body ideals, rights and power (1) Lack of support from friends and family (5)	The fitness centre as a social arena (5)	Lack of social connections (2) Lack of support from health authorities (1) Not fitting in (7)	Social connections (21)

According to the six categories on contextual factors, the main differences between AwPD and AwoPD were in the two first categories: (1) The setting, and (2) The fitness centre user characteristics, whereas the remaining four categories were more similar for the two groups. Differences and similarities between the groups are described below.

1. The fitness centre setting was viewed differently for the two groups. For AwPD, the barriers were reported as poor transportation options, an inappropriate interior fitness centre environment, and lack of adjustable exercise equipment, while the facilitators focused on means to overcome these barriers, especially for wheelchair users. For AwoPD, the focus was on easily accessible locations and flexible opening hours, along with a pleasant, clean environment and up-to-date equipment.

2. The fitness centre user characteristics also differed between groups. For the AwPD, most papers described barriers, such as not knowing the possibilities for exercising (e.g., where and when), the high cost, and negative feelings towards exercising in fitness centres. Only two papers [59,60] represented facilitators associated with exercising in fitness centres. These studies investigated AwPD in the process of undertaking education to become a fitness instructor, and no studies described the facilitators for the disabled participant exercising to maintain/improve fitness at a recreational athlete level (e.g., 0–2 times a week). This reveals a gap in the descriptions of AwPD and their reflections, wishes, and experiences of exercising in fitness centres. In contrast, for AwoPD, a large number of papers addressed facilitators (such as motivational factors) for fitness centre participation, for different subgroups, such as older people, men/women, and former/current users. Furthermore, few papers [71,113,123] uncovered barriers to fitness centre participation of people who are non-users, such as a dislike of the fitness centre culture or not having the time or motivation to exercise.

3. The fitness instructor/staff characteristics were viewed similarly in both groups, as they both preferred competent instructors with good social skills. One of the minor differences was that AwPD wished that instructors had professional skills to adapt/adjust their exercise programs, whereas AwoPD preferred a motivating instructor with a fit appearance (muscular, slender, and nice-looking). In both groups, the lack of skilled instructors was a barrier, while instructors with a solid background and excellent exercise skills were clear facilitators, together with having good social and communication skills.

4. The fitness centre user–instructor/management relationship did not differ much between the groups. They both favoured comfortable and welcoming fitness environments and positive interactions with instructors/management, but there were differences in the detailed descriptions. AwPD focused on the fitness centre not being actively inclusive, in addition to negative staff attitudes with unprofessional assistance. Facilitators were therefore characterised as an actual inclusive and tolerant environment with professional guidance from instructors. AwoPD focused on the negative attitudes and unachievable body ideals as barriers, leading to a feeling of not fitting into the fitness centre environment, while the facilitators included a pleasant atmosphere combined with professional, motivating, and fun instructors.

5. The fitness/exercise characteristics was the category with the fewest papers, but with considerable similarity across groups. Common to both groups was a focus on their individual needs; consequently, they requested fitness classes tailored to the type and level of physical condition/disabilities, and both groups requested help with the exercises. AwPD lacked access to tailored adapted classes and programs in general and had concerns with requesting assistance. For AwoPD, equipment-based exercising was perceived as boring and they preferred more fun and motivating exercises instead, e.g., fitness classes or individually tailored programs to achieve their goals and improve motivation.

6. Other relationships (relationships with other fitness centre users) showed some similarity across groups, as positive social connections were favoured among both groups. AwPD focused mainly on the negative interactions, e.g., stigma or negotiation about body ideals, while AwoPD focused on the limited social relationships and the experience of not fitting in (feeling of not being part of the community) as being barriers. In terms of facilitators, both groups found social relationships necessary and characterised the fitness centre as being a place to meet new people, peers, and even role models. Social relationships were further reported as essential for fitness centre-based exercise adherence for AwoPD.

## 4. Discussion

We identified 102 papers, with only one-quarter of the papers dealing with AwPD. Differences in identified barriers or facilitators between the two groups were seen in the fitness centre setting and the fitness centre user characteristics. AwPD mainly reported barriers related to inaccessibility and negative feelings towards exercising in fitness centres, whereas AwoPD mainly reported facilitators, such as individual motivational factors and the benefits of exercising. Large similarities between the two groups were seen in the remaining four categories. This scoping review is novel. To the best of our knowledge, this is the first time that the barriers and facilitators have been assessed for both AwPD and AwoPD, making a comparison between groups possible.

The current results are almost in line with a recent scoping review on gym-based exercise engagement among people with physical disabilities [142], as the reported barriers were lack of gym accessibility, oppressive attitudes within gyms, and also lack of social support during exercising, while the facilitators were reported to be enhanced opportunities to interact with others in the gym settings. That review included 15 papers, and only three of those were included in the current scoping review due to its narrow scope of fitness centre settings, compared with a broader scope in a variety of leisure time and fitness settings.

This focus on barriers to physical activity and lack of representation of AwPD in fitness centres is not new, and during the last two decades, several publications have tried to address the issue [11,47,56,60,62,65,143]. However, this issue still seems to persist as the needs of AwPD are still not being met, with many barriers still present—poor accessibility being the most dominant. Very few reviews about AwPD within the fitness centre setting were found, with one about measurement properties of instruments for assessing accessibility [144], another about accessibility in fitness centres [26], and finally the one mentioned above about gym-based exercise participation, which had a broader scope than fitness centres [142]. This underlines the relevance of our study, providing an overview of a broad spectrum of both barriers and facilitators. In particular, knowledge about wishes, desires, and preferences for exercising in fitness centres with a focus on the facilitators is important to provide guidance for the fitness centres and their users with disabilities.

An interesting point was that the AwPD group was reported as one homogeneous group, while they actually varied in many aspects depending on their level of physical disability and origin (congenital or acquired). In contrast, AwoPD was reported as a heterogeneous group, differing in gender, age, amount of fitness centre experience, pregnancy, obesity, etc. One of the reasons may be due to the group of AwPD being smaller than the group of AwoPD, combined with the limited knowledge of fitness centre participation for AwPD in general, and with only two papers reporting experiences from the perspective of AwPD themselves [59,60]. Moreover, these two papers included participants from an educational program for AwPD who aspired to become gym instructors, limiting the representativeness of AwPD in general.

The most commonly reported barriers differed between the two groups. For AwPD, the most common barrier included all aspects of physical fitness centre inaccessibility, such as inadequate transportation options and non-adjustable exercise equipment, which mirror results from a recent scoping review on gym settings [142]. For AwoPD, the barriers were lack of motivation or adherence to exercise in fitness centres. An explanation for this difference may be that for many AwPD, the physical barriers were the first obstacles determining participation in fitness centre exercising, meaning they did not have much experience with fitness centre participation beyond the front door. For AwoPD, the barriers were related to the individual (lack of time or interest, lack of knowledge, and negative aspects towards the fitness culture), which ultimately determined whether they entered the fitness centre.

The most commonly reported facilitators differed between groups. Among AwPD, the primary facilitator, not surprisingly, was the positive side of accessibility, namely, an accessible environment/universal design/adjustable fitness equipment. Facilitators for AwoPD were related to a comfortable environment in the fitness centre, as well as the opportunity to become healthier and improve body appearance and well-being. It therefore seems that the majority of barriers and facilitators for AwoPD within the categories (1) The fitness centre setting and (2) The fitness centre user characteristics may also be applicable to AwPD, as they relate to the individual person and not the disability.

Importantly, AwPD experienced negative feelings related to being in the fitness centre, such as respect for users’ dignity, perceptions of otherness, feeling a burden, or losing autonomy [51]. These barriers were unique to AwPD and are often referred to as direct and indirect psycho-emotional disablism [145], where direct psycho-emotional disablism (‘acts of invalidation’) is the negative interaction (verbal and non-verbal) that occurs with other people, and indirect psycho-emotional disablism is the negative influence of structural (physical) barriers on AwPD, resulting in the negative feelings related to exclusion and discrimination [145]. AwPD experience barriers related to their disability and not to them as individuals, and as described, facilitators are often reported as the opposite of the barriers; i.e., good accessibility. This was contrary to AwoPD, where barriers and facilitators were related to them as individual people and their specific interests, motivations, goals, etc. Therefore, for AwoPD, facilitators were not just the opposite of the barriers identified within the same contextual factors. However, due to the few papers concerning AwPD, more research is needed on how interactions with other fitness users act as a barrier or facilitator for participation in fitness centres.

As mentioned above, there were several similarities across the two groups. Generally, they reported facilitators as competent instructors, comfortable and welcoming fitness centre environments, cheap membership, exercising at their preferred type and level, and good social connections during exercising. Overall, both groups reported fitness centres that could meet their individual specific needs as facilitators, whereas differences occurred on how these needs should be met. AwPD were seeking skills from an instructor who could adjust their exercises to suit their specific needs, and AwoPD preferred instructors who could motivate, make exercising fun, and make them commit to exercising. The current findings are mostly in line with a recent systematic review, summarising that facilitators of adherence to exercise referral schemes were social support (from professionals, family/friends and peers), accessible settings (central location and good transportation), individually tailored and varied programs, flexible attendance hours, and perceived benefits of physical and mental health [146].

The included number of papers differed markedly between the groups. This was surprising, as exercising in fitness centres is a more complex task for AwPD than AwoPD, and therefore a higher number of papers involving AwPD with different diagnoses/subgroups was expected. However, this unbalanced distribution in papers may be due to the fact that AwPD is a marginal group in fitness centres and therefore little knowledge about this group is still available.

The included papers further differed between groups on type (scientific/grey) and main focus (barriers/facilitators). Guidelines on how to overcome physical barriers (e.g., by universal design) and plan for the exercise session were only reported in studies on AwPD [49,52,53,56,64,65]. These guidelines varied in size and scientific quality, and some were even more related to general sports facilities than to fitness centres [49,65]. In line with recommendations from the included six guidelines, a recent systematic review [26] summarised that both physical and system access barriers (e.g., policies, programs, and professional behaviour) limit AwPD in using fitness centres. Furthermore, it was reported that accessibility to fitness centres is very dependent on the legislation underpinning building compliance, which seems to still present the minimum standards [26].

In contrast to the focus on barriers for AwPD, papers on AwoPD mostly focused on facilitators of exercising in fitness centres, and some of them investigated the motivational factors for economic and/or health promotion benefits [68,77,107,109]. Surprisingly, despite the large number of papers investigating the facilitators for AwoPD, no reviews within this area were identified.

### Method—Limitations and Strengths

The limitations of the study were primarily related to the selected databases, as other databases could have been included (e.g., ProQuest, Cochrane Library, AMED, Web of Science, PEDro, or OTseeker). However, many of these databases are small or with a very narrow scope, and moreover, a high number of duplicates were already present within the six selected research databases, when using database-specific subheadings. Another limitation was that we did not screen the reference lists of all the included papers for additional records, as stated in the a priori protocol. This was only performed for the ‘grey literature’. Citation searching of our included records from the databases may thus have increased the number of records. However, the broad search across databases and the large number of screened records are anticipated to compensate for that. Finally, the narrow range of the current scoping review, limited to fitness centres for adults, has led to exclusion of studies on physical activity/general exercising and sports participation, studies related to the healthcare sector and the recreation sector, in addition to studies with mixed groups of children and adults. The literature search also identified references from the year 1995 onwards, resulting in a broad time span, in which fitness centre culture and a customer base may have developed.

One key strength of the current study was the selection of a scoping review rather than a systematic review as the method, which is especially appropriate for this research question due to its broader approach [30,31]. Moreover, we included all types of literature, as recommended for scoping reviews [32]. Another strength of the study is that recommended guidelines for conducting and reporting scoping reviews were followed accurately [38], and the method with procedures was presented in an a priori published protocol [37], including a comprehensive literature search, study selection, and data-extraction performed by two reviewers independently.

Further, the use of the Di Blasi framework was suitable for this scoping review. The Di Blasi framework [39], used to categorise the barriers and facilitators, was slightly modified to target the context of the fitness centres, with the addition of a sixth category to accommodate the fact that exercising in a fitness centre means interacting with other users and staff, in contrast to one patient receiving treatment from a healthcare practitioner. We are aware of the Di Blasi framework [39] originating from a rehabilitation/healthcare setting (practitioner–patient interaction). Whether the transition to a fitness centre setting (staff–fitness centre user interactions) has influenced our analysis and results remains unknown, since aspects such as societal structures, culture, and economics may have an influence. Alternative guidelines or frameworks could have been selected to categorise the identified barriers and facilitators, but the broader terminology in the Di Blasi framework encompassed more aspects of fitness centres (covered by the six categories of contextual factors) than, for example, a checklist for only accessibility [53] or guidelines from organisations or legislation [56,65,147]. The classification of both the barriers and facilitators using the modified Di Blasi framework facilitated their meaningful distribution over the six categories and was found to be comprehensive enough.

## 5. Conclusions

Based on the six contextual factor categories for exercising in fitness centres, the facilitators and barriers associated with fitness centre use differed between AwPD and AwoPD. The main focus for AwPD was on barriers due to inaccessibility, whereas for AwoPD, it was on facilitators such as motivational factors and benefits of exercising. Similarities were seen in the barriers/facilitators regarding the presence of skilled instructors, a comfortable and welcoming fitness centre environment, opportunity to exercise at the preferred type and level, and good social connections during exercising. However, the details on these facilitators/barriers differed between groups. For AwPD, the barriers/facilitators were often related to their disabilities and not themselves as individuals, whereas for AwoPD, the barriers/facilitators were related to the individual and their personal wishes, desires, and preferences for exercise.

Since only one-quarter of the studies focused on AwPD, more studies on the actual experiences (barriers, facilitators) of AwPD regarding fitness centre use are especially needed, whereas the main barrier—inaccessibility—is fairly well described. In particular, knowledge on how interactions with AwPD, instructors/staff, and other users can be optimised is lacking. Further, although motivational factors and preferences were reported as important for AwoPD, similarities and differences in relation to AwPD on these contextual factors need more investigation. Finally, more research is needed on the barriers and facilitators for non-users, to attract new members of AwPD to exercising in fitness centres together with AwoPD.

## Figures and Tables

**Table 1:** A modified version of the Di Blasi framework of contextual factors. The six categories were used to categorise the barriers to, and facilitators of, exercising in fitness centres in this review.

Context Factor Categories		Description
1	The Fitness Centre Setting	The physical environment in the specific fitness centre/gym, e.g., surrounding area, buildings, room arrangement, and fitness equipment.
2	The Fitness Centre User Characteristics	The ‘personal factors’ according to ICF [40] combined with their physical ability, e.g., bodily performance and the individual participant’s opinions and feelings.
3	The Fitness Instructor’s/Staff Characteristics	The front-line personnel in the fitness centre and their qualifications, e.g., knowledge, education, appearance, communication skills, and courtesy, etc.
4	The Fitness Centre User—Instructor/Management Relationship	The direct or indirect interaction between the participant and the instructor/management who represent the fitness centre as a whole with respect to personal relations, teaching, and prejudices when interacting as a representative of the specific fitness centre, together with the rules, policies, membership terms and conditions, artefacts, culture, and the atmosphere of the place.
5	The Fitness/Exercise Characteristics	The different types of fitness exercises and how they are performed, e.g., individual exercising, types of classes, planning, specific exercises, etc.
6	Other Relationships	The relationship or direct and indirect interactions with other people than the staff in the fitness centre, e.g., strangers, familiar faces, friends and family, or personal assistants.

## Data Availability

The data that support the findings of this study are available from the corresponding author upon reasonable request.

## Supplementary Materials

The following are available online at https://www.mdpi.com/article/10.3390/ijerph18147341/s1, S1: Search strings of the six databases, S2: Excel sheet for data extraction, Table S3: Excel sheet for data extraction.

## Abbreviations

AwPD	Adults with physical disabilities
AwoPD	Adults without physical disabilities
PCC mnemonic	Population, Concept and Context
WHO	World Health Organization
