# Supplementary material for: Barriers to, and Facilitators of, Exercising in Fitness Centres among Adults with and without Physical Disabilities: A Scoping Review
Source: Int J Environ Res Public Health. 2021 Jul 9;18(14):7341. doi: 10.3390/ijerph18147341 (PMC8304633; doi:10.3390/ijerph18147341)
Supplement: Supplementary file 1 [file ijerph-18-07341-s001.zip › Sup1_searchstrings.pdf]

Supplementary 1:
Database searches performed October 15th 2018 and May 19th 2020.

Medline via Pubmed:
((((((((((((((((("Fitness Centers"[Mesh]) OR "fitness center") OR "fitness centers") OR "fitness centre") OR "fitness centres") OR gym) OR gyms) OR "fitness gym") OR "fitness gyms") OR "health club") OR "health clubs") OR "fitness club") OR "fitness clubs") OR "fitness facility") OR "fitness facilities") OR "fitness academy") OR "fitness academies") OR "fitness institute") OR "fitness institutes")) AND (((((("Adult"[Mesh]) OR adult) OR adults) OR "over 18 years") OR "+ 18 years") OR ("18 years and over"))

Scopus via Elsevier:
((TITLE-ABS-KEY (adults) OR TITLE-ABS-KEY (adult) OR TITLE-ABS-KEY ("over 18 years") OR TITLE-ABS-KEY ("+ 18 years") OR TITLE-ABS-KEY ("18 years and over"))) AND ((TITLE-ABS-KEY ("fitness center") OR TITLE-ABS-KEY ("fitness centre") OR TITLE-ABS-KEY ("gym") OR TITLE-ABS-KEY ("Fitness gym") OR TITLE-ABS-KEY ("health club") OR TITLE-ABS-KEY ("fitness facility") OR TITLE-ABS-KEY ("fitness academy") OR TITLE-ABS-KEY ("fitness institute")))

SportDiscus via EBSCO:
(DE "PHYSICAL fitness centers" OR "fitness center" OR "fitness centers" OR "fitness centre" OR "fitness centres" OR gym OR gyms OR "fitness gym" OR "fitness gyms" OR "health club" OR "health clubs" OR "fitness facility" OR "fitness facilities" OR "fitness academy" OR "fitness academies" OR "fitness institute" OR "fitness institutes") AND (adult OR adults OR "over 18 years" OR "+ 18 years" OR ("18 years or over")

Cinahl via EBSCO:
(MM "Fitness Centers" OR "fitness center" OR "fitness centers" OR "fitness centre" OR "fitness centres" OR gym OR gyms OR "fitness gym" OR "fitness gyms" OR "health club" OR "health clubs" OR "fitness facility" OR "fitness facilities" OR "fitness academy" OR "fitness academies" OR "fitness institute" OR "fitness institutes") AND (MM "Adult" OR adult OR adults OR "over 18 years" OR "+ 18 years" OR "18 years or over")

PsycInfo via Ovid:
(("fitness center" or "fitness centers" or "fitness centre" or "fitness centres" or "gym" or "gyms" or "fitness gym" or "fitness gyms" or "health club" or "health clubs" or "fitness facility" or "fitness facilities" or "fitness academy" or "fitness academies" or "fitness institute" or "fitness institutes") and (adult or adults or "over 18 years" or "+ 18 years" or "18 years and over")).ab,hw,id,ti.

Embase via Ovid:*
(("fitness center" or "fitness centers" or "fitness centre" or "fitness centres" or "gym" or "gyms" or "fitness gym" or "fitness gyms" or "health club" or "health clubs" or "fitness facility" or "fitness facilities" or "fitness academy" or "fitness academies" or "fitness institute" or "fitness institutes") and (adult or adults or "over 18 years" or "+ 18 years" or "18 years and over")).ab,hw,id,ti

* id=key concepts. A small change had been made in the database between the searches, so this field was not used for the search in 2020.
