# Supplementary material for: Barriers to, and Facilitators of, Exercising in Fitness Centres among Adults with and without Physical Disabilities: A Scoping Review
Source: Int J Environ Res Public Health. 2021 Jul 9;18(14):7341. doi: 10.3390/ijerph18147341 (PMC8304633; doi:10.3390/ijerph18147341)
Supplement: Supplementary file 1 [file ijerph-18-07341-s001.zip › Sup3_102_articles.pdf]

Table S3: The 102 papers included in the scoping review. Alphabetic order by first author. Papers concerning people with disability are grey.

Ref 102	Author(s)	Year	Title	Origin	Population	Type of publication	
				Were the study was conducted		Research (qual, quan, mixed)	Grey
[1]	Allen J. E.	2001	An exercise in frustration	USA, Los Angeles, California	1 person with post-polio syndrome, 1 Parkinson patient + researchers and scientists (talking about people with disability in general)		Newspaper article
[2]	Anderson et al.	2017	Exercise facilities for neurologically disabled populations - Perceptions from the fitness industry	UK	Fitness facility managers (talking about people with neurological disabilities)	Mixed	
[3]	Andreasson et al.	2016	Keeping Death at Bay through Health Negotiation: Older Adults' Understanding of Health and Life within Gym and Fitness Culture	Sweden	24 older adult gym-goers (10 men, 14 women). Age between 63 and 83 years (median 70).	Qual	
[4]	Arbour-Nicitopoulos et al.	2011	Universal Accessibility of "Accessible" Fitness and Recreational Facilities for Persons With Mobility Disabilities	Canada	None	Quan	
[5]	Asserhøj	2017	Danes' fitness habits and use of commercial sports offers [Danskernes fitnessvaner og brug af kommercielle idrætstilbud]	Denmark	3914 Danish adults >16 years		Report
[6]	Athanasopoulou et al.	2011	Consumer Behaviour in Fitness Centers: A Typologi of Customers	Greece	350 fitness center users (61% women, 39% men) mainly age 24-50		Conference paper
[7]	Berke et al.	2006	Distance as a barrier to using a fitness-program benefit for managed Medicare enrollees	USA, Washington	total sample size of 8,162 people >65 years. A total of 1,728 participants in the unstructured program were matched with 4,838 nonparticipants, and 421 participants in the structured program were matched with 1,175 nonparticipants.	Quan	
[8]	Bethancourt et al.	2014	Barriers to and facilitators of physical activity program use among older adults	USA, Washington	Participants (N = 52), ages 66 to 78, were primarily Caucasian, retired, married, had a college or graduate degree, had good to excellent self-rated health, and had high self-reported levels of PA	Qual	
[9]	Brown et al.	2017	Comparing current fitness center members' perceptions of the motivational climate with non-members	USA	N=657, age 22-76 (65% female, 35% male) never users (n = 138), former users (n = 213) and current users (n = 306)	Mixed	
[10]	Brown et al.	2014	Faculty/staff perceptions of a free campus fitness facility	USA, large Southern university	N=657, age 22-76, (65% female, 35% male) never users (n = 138), former users (n = 213) and current users (n = 306)	Mixed	
[11]	Brownfield	2002	Setting his own pace in the gym rat race	USA, Los Angeles, California	The author a 36 year old male, new fitness user.		Newspaper article
[12]	Calder et al.	2018	The accessibility of fitness centers for people with disabilities: A systematic review	New Zealand - (fitness centers located in USA 10, Kuwait 2, Canada 1, Singapore 1)	None (fitness facilities)	Systematic review	
[13]	Campos et al.	2017	Fitness participants perceived quality by age and practiced activity	Portugal, Coimbra municipality	622 group exercise women, minimum 18 years of age.	Quan,	
[14]	Caudwell et al.	2016	The Effect of Men's Body Attitudes and Motivation for Gym Attendance	Australia	100 male participants age range of 18–68 years, who attended a gym or fitness centre frequently.	Quan,	
[15]	Choitz et al.	2010	Urban Fitness Centers: Removing Barriers to Promote Exercise in Underserved Communities	USA, Pennsylvania	802 adults, new members, 78% women, mean 54 years old, mean BMI 32 (obese).	Quan,	
[16]	Courneya et al.	1997	A Simple Reinforcement Strategy for Increasing Attendance at a Fitness Facility	Canada	300 randomly selected paying members. Age 21-60.	Quan	
[17]	Cyr et al.	2019	Might plight: The social anxiety felt by men in the weightlifting environment	Canada, Southwestern Ontario region	299 male gym-goers, working out in the weight lifting environment		Magazine article
[18]	Dogan	2015	Training at the Gym, Training for Life: Creating Better Versions of the Self Through Exercise	UK, London	32 active gym members of whom 20 were women and 12 men, all students or working adults, ages 23 to 69	Qual	
[19]	Dolbow et al.	2015	Accommodation of wheelchair-reliant individuals by community fitness facilities	USA, Mississippi, (Hattiesburg)	None	Quan	
[20]	Emeterio et al.	2019	Prediction of abandonment in Spanish fitness centres	Spain, Zaragoza and Tudela	14,522 customers of 3 sports centres	Quan	
[21]	Evans et al.	2019	Groupness perceptions and basic need satisfaction: Perceptions of fitness groups and experiences within club environments	USA and New Zealand	293 exercisers (mean age 35.93, *SD* 11.44, 78% female, 22% male)	Quan	
[22]	Evans et al.	2019	Living for Today or Tomorrow? Self-Regulation amidst Proximal or Distal Exercise Outcomes	USA	Study 4; new members at a commercial gym (N = 210)	Quan	
[23]	Faulkner et al.	2019	Examining the use of loyalty point incentives to encourage health and fitness centre participation	Canada, in Alberta, New Brunswick and Ontario	459.146 participants from fitness centres	Quan	

[24]	Fieril et al.	2014	Experiences of exercise during pregnancy among women who perform regular resistance training: A qualitative study	Sweden, Gothenburg	17 pregnant woman, regular exercisers	Qual	
[25]	Fredslund et al.	2019	Can the Easter break induce a long-term break of exercise routines? An analysis of Danish gym data using a regression discontinuity design	Denmark	1210 gym members. 63% women, 37% men Mean age 42.4 years.	Quan	
[26]	Fullerton et al.	2008	Survey of fitness facilities for individuals post-stroke in the Greater Toronto Area	Canada, Greater Toronto Area	Fitness facilities managers	Quan	
[27]	Gjestvang et al.	2019	Are changes in physical fitness, body composition and weight associated with exercise attendance and dropout among fitness club members? Longitudinal prospective study	Norway, Oslo	125 untrained new members, unequally men and women.	Quan	
[28]	Gross et al.	2013	Accessibility of fitness centres for people with disabilities in a region in North East Scotland	Scotland, north eastern part	None	Quan	
[29]	Harada et al.	2014	Perceived and objectively measured access to strength-training facilities and strength-training behavior.	Japan, Tokyo, Nerima & Kanuma	1051 persons, aged 40–69 years categorized into two groups: those who engaged in regular strength-training behavior (minimum 2 time a week) and those who did not.	Quan	
[30]	Heinrich et al.	2017	Mapping Coaches' Views of Participation in CrossFit to the Integrated Theory of Health Behavior Change and Sense of Community:	USA, the midt west	Participants (N=6) were head coaches/owners from six Midwest CrossFit affiliates.	Qual	
[31]	Hosek	1997	Self-motivation and exercise adherence in adult women	USA, Texas, Houston	Healthy female adults: 50 participants in the age range of 18 to 70. All participants were beginners to exercise programs or returning to exercise after non-activity for a period of at least one year.		Thesis
[32]	Hurley et al.	2012	Universal design of fitness equipment criteria to meet the new department of justice accessibility requirements	USA	None (fitness equipment)		Conference paper
[33]	Hurrell	1997	Factors Associated with Regular Exercise	USA, New York, Westchester County	450 adults (*59%, n=267 women and 41%, n = 183 men)* who belonged to the health club sponsoring the study.<br>The majority of the respondents *(76.8%, n =346)* were between *25* and *65* years of age.	Quan	
[34]	Inclusive Fitness Coalition	2015	Making Your Health & Fitness Center a Welcoming Facility	USA	None		Guideline
[35]	Jang et al.	2018	Factors influencing choice when enrolling at a fitness center	Korea, Seoul	283 participants enrolled in fitness centers (60.4% *n* = 171 men and 39.6% *n* = 112 women).<br>Age;<br>42.0% (*n* = 119) 20-29 years,<br>37.8% (*n* = 107) 30-39 years,<br>16.6% (*n* = 47) 40-49 years,<br>3.5% (*n* = 10) over 50 years.	Quan	
[36]	Jekauc et al.	2015	Prediction of attendance at fitness center: a comparison between the theory of planned behavior, the social cognitive theory, and the physical activity maintenance theory	Germany	Participants were 101 (48 males and 53 females) college students and members of a fitness center. Age ranged from 19 to 32 years	Quan	
[37]	Johnson et al.	2012	ADA Compliance and Accessibility of Fitness Facilities in Western Wisconsin	USA, Western Wisconsin	None	Quan	
[38]	Johnston et al.	2015	Understanding dignity: experiences of impairment in an exercise facility	Canada, Alberta?	21 adult fitness center users (11 women and 10 men) 19–65 years of age. They attended the fitness center to receive exercise support because of self-reported neurological (n = 14), sensory (n = 2), or mobility impairments (n = 5).	Qual	
[39]	Kailes	2008	Using a Fitness Center Does Not Have to be an Exercise in Frustration: Tips for People with Mobility and Visual Disabilities	USA, California, Pamona	None (aiming at people with mobility disabilities)		Guideline
[40]	Kaushal et al.	2015	Exercise habit formation in new gym members: a longitudinal study	Canada, British Columbia, Greater Victoria region	111 participants age of 18–65, and being a recent gym member, which was defined as someone who has joined a gym/recreation centre within the past 2 weeks.	Quan	
[41]	Kaushal et al.	2017	The role of habit in different phases of exercise	Canada	Participants (n = 181) were a sample of adults (18–65) who have been exercising for at least 1 year.	Quan	
[42]	Kirkegaard	2009	Part 2: Portray of the active fitness customers: motives for training, satisfaction and self-reported health [Delrapport 2: Portræt af de aktive fitnesskunder: træningsmotiver, tilfredshed og selvvurderet sundhed]	Denmark	4747 adult active fitness members		Report

[43]	Kirkegaard et al.	2010	Part 5: Fitness culture between sports club and business: active fitness users motives for training, satisfaction and self-reported health [Delrapport 5: Fitnesskultur mellem forening og forretning: aktive fitnessudøveres træningsmotiver, tilfredshed og selvvurderede sundhed]	Denmark	Active fitness users; 1.842 members from non-profit fitness centres and 4.623 from commerciel fitness centres		Report
[44]	Klein	2002	Make a Positive Connection	USA	Authors perspective as a former obese fitness user and now as s personal trainer.		Magazine article
[45]	Kruisselbrink et al.	2004	Influence of Same-Sex and Mixed-Sex Exercise Settings on the Social Physique Anxiety and Exercise Intentions of Males and Females	Canada, eastern part	61 women and 35 men, members of a coed fitness facility.	Quan	
[46]	Larson et al.	2017	You can't always get what you want: expectations, outcomes, and adherence of new exercisers	Canada	18 participants (10 female, eight male) aged 35–64 years.	Qual	
[47]	León-Quismondo et al.	2020	Service Perceptions in Fitness Centers: IPA Approach by Gender and Age	Spain, Madrid	414 fitness members 173 women and 241 men) with a mean age of 32.33 years.	Quan	
[48]	Lockett	2011	Information Package on AIMFREE Accessibility Instruments Measuring Fitness and Recreation Environments	USA	None		Guideline
[49]	Lopez-Fernandez et al.	2018	A Weekly Structured Physical Activity Program Enhances Short-term Retention Of Middle-aged Adult Fitness Centre Users	Spain	80 inactive middle-aged adults		Conference poster
[50]	Lübcke et al.	2012	Older Adults' Perceptions of Exercising in a Senior Gym	Sweden, Stockholm	eight elderly, three men and five women between ages 65 and 81. 3–6 months of exercise in the center.	Qual	
[51]	Malek et al.	2002	Importance of Health Science Education for Personal Fitness Trainers	USA, California, Inland Empire area	115 health fitness professionals (61 men and 54 women), ages 20 to 54. Mean age 30.1. Coming from Independent health club, Corporate-owned health club, self-imployed or College facility.	Quan	
[52]	Manning et al.	2019	Adopting a functional fitness approach to membership	USA, North Carolina	None (the perspective of a fitness center owner)		Magazine article
[53]	Martin et al.	2005	Exercise and older women's wellbeing	Australia	10 women. 50 years of age or over who have participated in physical activity for a minimum of thirty minutes on at least three days of the week for the past two years.	Qual	
[54]	McDonnell	2002	Family-friendly locker rooms: as the demographics in your facility change to include more families and older adults, catering to their locker room needs may be to your advantage	USA	None		Magazine article
[55]	Melton et al.	2010	The current state of personal training: managers' perspectives	USA, North Carolina	11 managers of personal trainers (survey data) only 9 of them for the focus group	Qual	
[56]	Melton et al.	2008	The Current State of Personal Training: An Industry Perspective of Personal Trainers in a Small Southeast Community:	USA, North Carolina	11 personal trainers	Qual	
[57]	Middelkamp et al.	2016	The Effects of Two Self-Regulation Interventions to Increase Self-Efficacy and Group Exercise Behavior in Fitness Clubs	Netherlands	122 participants (67% women). Agerange 18-70 years, mean age 42. No membership of a fitness club for the past 6 month.	Quan	
[58]	Milner	2005	Equipping your fitness centre for older adults	Canada	None (fitness equipment for older adults)		Magazine article
[59]	Mullen et al.	2010	Age, gender, and fitness club membership: Factors related to initial involvement and sustained participation	USA, Virginia	Participants N = 326 (71% female), were recruited via a national online research and marketing firm. Ages<br>Young (25-34; N = 58),<br>middle aged (35-54; N = 149),<br>older adults (55 and over; N = 119).	Quan	
[60]	Nary et al.	2000	Accessibility of Fitness Facilities for Persons with Physical Disabilities Using Wheelchairs	USA, Kansas, Topeka	None	Quan	
[61]	North Carolina Office on Disability and Health	2008	Removing barriers to Health Clubs and Fitness Facilities - A guide for Accommodating All Members, Including People with Disabilities and Older Adults	USA, North Carolina	None		Guideline
[62]	Østerlund et al.	2010	Fitnesscentre i firmaidrætten - portræt af de aktive medlemmer: træningsmotiver, tilfredshed og selvvurderet sundhed.	Denmark	226 adult fitness users, mean age 40 years. 53% women, 47% men		Report
[63]	Østerlund et al.	2010	Foreningsfitness – portræt af de aktive medlemmer: træningsmotiver, tilfredshed og selvvurderet sundhed	Denmark	1616 active fitness users. 55% women and 45% men. Mean age 44.		Report

[64]	Pettigrew et al.	2018	A typology of factors influencing seniors' participation in strength training in gyms and fitness centers	Australia, Western part and Perth	service providers (n=18 instructors, n=24 center managers)<br>4 focus groups<br>health/community care practitioners (n=8), individuals who advise on and implement health policies relating to physical activity (n=5), seniors +60 years (n = 13, n = 11).	Qual	
[65]	Rabiee et al.	2015	Gym for Free: The short-term impact of an innovative public health policy on the health and wellbeing of residents in a deprived constituency in Birmingham, UK	UK, Birmingham	257 users. 144 (56%) men and 113 (44%) women responded to the questionnaire.<br><br>9 participated in three focus groups: 2 men and 7 women.<br>8 staff members from the leisure centres formed the fourth focus group: 6 women and 2 men.	Mixed	
[66]	Rasmussen et al.	2018	An explorative evaluation study of the mechanisms underlying a community-based fitness centre in Denmark – Why do residents participate and keep up the healthy activities?	Denmark, Ålborg	5 instructors and 5 fitness users (3 men and 2 women in each group)	Mixed	
[67]	Rauworth	2006	Designing a fitness facility for all	USA	None		Magazine article
[68]	Rekieta	2002	Exercise relapse prevention: The efficacy of a motivational interview intervention	USA, Memphis	87 adult members (59% women) who had joined the facility within the previous 15 day and were currently exercising less than 5 days per month.		Thesis
[69]	Richardson et al.	2017	Crossing boundaries: The perceived impact of disabled fitness instructors in the gym	UK	10 disabled persons (5 male and 5 female), who were becoming gym instructors. Age ranged from 23 to 60 with an average age of 40. Eight participants had acquired impairments and two were congenital.	Qual	
[70]	Richardson et al.	2017	Collective Stories of Exercise: Making Sense of Gym Experiences With Disabled Peers	UK	18 disabled participants enrolled in a gym instructor training program were recruited; 10 were male and 8 female. The ages of participants ranged between 23 and 60 years, average 40 years. 15 individuals had acquired their impairments in their teenage years or adulthood, and 3 were congenital or became impaired in early childhood.	Qual	
[71]	Richardson et al.	2017	Disability and the gym: experiences, barriers and facilitators of gym use for individuals with physical disabilities	UK	21 disabled participants enrolled in a gym instructor program were recruited; 13 were male, and 8 were female. Age-range between 23 and 60 years, average 40. 18 individuals had acquired their disabilities and 3 were born with them.	Qual	
[72]	Riley et al.	2008	A conceptual framework for improving the accessibility of fitness and recreation facilities for people with disabilities	USA, Chicago	None	Review/opinion paper	
[73]	Rimmer et al.	2004	Physical activity participation among persons with disabilities	USA, participants from 10 regions (Atlanta, Baltimore, Berkeley, Boise, Boston, Chicago, Denver, Houston, Kansas City, and Syracuse)	A total of 42 persons.<br>The four focus groups included:<br>(1) people with disabilities<br>(2) architects<br>(3) fitness/recreation professionals<br>(4) city planners and park district managers.	Qual	
[74]	Rimmer et al.	2017	Fitness facilities still lack accessibility for people with disabilities	USA, 10 states	None	Quan	
[75]	Riseth et al.	2019	Lon-term members' use of fitness centers: A qualitative study	Norway, Trondheim	21 long-term members (> 2 years) membership from 2-20 years<br>11 females and 10 males average age was 43 years (range 20–71 years).	Qual	
[76]	Rodrigues et al.	2019	Have you been exercising lately? Testing the role of past behavior on exercise adherence	Portugal	293 exercisers (female=166; male=127) age 18 – 65 years (M=36.57±SD=11.25)	Quan	
[77]	Rodrigues et al.	2019	The role of dark-side of motivation and intention to continue in exercise: A self-determination theory approach	Portugal	544 (294 female; 250 male) gym exercisers aged between 18 and 58 years (M = 35.00; SD = 11.57) exercise experience ranged from 3 to 120 months (M = 47.41; SD = 7.54	Quan	
[78]	Rodrigues et al.	2020	The bright and dark sides of motivation as predictors of enjoyment, intention, and exercise persistence	Portugal	575 gym exercisers (female = 230) aged between 18 and 65 years (M = 34.07; SD = 11.47) with at least 6 months of regular exercise practice	Quan	
[79]	Schmidt et al.	2019	"Kicked out into the real world": prostate cancer patients' experiences with transitioning from hospital-based supervised exercise to unsupervised exercise in the community	Denmark	29 men, prostata-cancer-survivers. Median 71 inter quartile range 67–74.	Qual	
[80]	Schvey et al.	2017	The experience of weight stigma among gym members with overweight and obesity	USA, Major cities from different geographical regions	389 gym-users, men (25%) and women (75%) with overweight (25 ≤ BMI <30; 26%) and obesity (BMI ≥ 30; 74%) participated. Average age was 32.98 ± 11.29 years, and mean BMI was 35.59 ± 7.66.	Quan	
[81]	Schwetschenau et al.	2008	Barriers to physical activity in an on-site corporate fitness center	USA, Midwestern part	88 employees. The sample of respondents was 74% female, with a mean age of 37 years (*SD* ± 10.21). Fifty-eight percent of respondents were members of the on-site fitness center	Quan	

[82]	Souza et al.	2018	Perspectives on Increasing Positive Attitudes Toward Larger Members in Fitness Centers	USA	A convenience sample n=155 (120 female, 31 male, and 4 “other” gender participants) Participants identified as a current member, past member or professional employed in a fitness center.	Mixed	
[83]	Sperandei et al.	2016	Adherence to physical activity in an unsupervised setting: Explanatory variables for high attrition rates among fitness center members	Brazil, Rio de Janeiro	5240 individuals (58.8 female), equivalent to all new clients who registered for the first time during the period between January 2005 and June 2014. Age: Up to 25 years 26.7%, 26–35 year 50.1%, 36 years and older 23.2%	Quan	
[84]	Springer et al.	2013	Maintaining physical activity over time: The importance of basic psychological need satisfaction in developing the physically active self	USA, midsized Midwestern city	12 participants (7male, 5 female; age range 29 to 73 years; average = 54 years) who were members at a health/fitness facility and had been regularly active at recommended levels for at least 3 years.	Qual	
[85]	Stein	2003	Bodywork. A swing toward families: gyms are taking a fresh look at classes for kids and parents to encourage old and young to plan their exercise time together	USA, California, Los Angeles, Hollywood	None		Newspaper article
[86]	Stein	2003	Bodywork. Bracing for the attack of the gym 'newbies'	USA, California, Los Angeles	None		Newspaper article
[87]	Stenson	2005	Workout partners: health clubs and videos are incorporating kids into routines so time-strapped parents can Strollercize, lift weights or do yoga with children in tow	USA, California, Los Angeles	None		Newspaper article
[88]	Stewart et al.	2014	The significance of critical incidents in explaining gym use amongst adult populations	Australia, Melbourne	10 gym-users (6 male, 4 female) were employed in professional occupations, or were university students. Their ages ranged from 23 to 64, mean age 44.	Qual	
[89]	Strelsand	2007	No Six-Packs Here, Please	USA	A 58 year old woman		Magazine article
[90]	Swoyer	2008	Equality of fitness centers: are all fitness centers created equal?	USA	None		Magazine article
[91]	Thomson et al.	2016	An exploration into the development of motivation to exercise in a group of male UK regular gym users	UK, London	28 male regular gym users (aged > 21 years) - 5 in the interviews	Mixed	
[92]	Tolle et al.	2018	Establishing the NeuroRecovery Network Community Fitness and Wellness facilities: multi-site fitness facilities provide activity-based interventions and assessments for evidence-based functional gains in neurologic disorders	USA	people with spinal cord injury and other physical disabilities		Guideline
[93]	Ulseth	2008	New Opportunities - Complex Motivations: Gender Differences in Motivation for Physical Activity in the Context of Sports Clubs and Fitness Centers	Norway	Fitness center users n= 1585 (30% men, 70% women	Quan	
[94]	Unger et al.	1995	Social relationships and physical activity in health club members	USA, California	200 members of at health club, age 21-79	Quan	
[95]	United states access board	2003	Accessible sports facilities	USA	None		Guideline
[96]	van der Swaluw et al.	2018	Commitment Lotteries Promote Physical Activity Among Overweight Adults—A Cluster Randomized Trial	Netherlands	163 overweight participants	Quan	
[97]	van der Swaluw et al.	2018	Physical activity after commitment lotteries: examining long-term results in a cluster randomized trial	Netherlands	163 overweight participants	Quan	
[98]	Vlachopoulos et al.	2007	A prospective study of the relationships of autonomy, competence, and relatedness with exercise attendance, adherence, and dropout	Greece	228 exercise participants. (47.4% male, 52.6% female)	Quan	
[99]	Wayment et al.	2017	Sharing a personal trainer: Personal and social benefits of individualized, small-group training	USA, southwestern part	98 regular exercisers (64 women and 32 men). Age range for the subjects was 19–78 years, mean 46.52 years. Average membership time 2 years.	Mixed	
[100]	Whiteman-Sandland et al.	2018	The role of social capital and community belongingness for exercise adherence: An exploratory study of the CrossFit gym model	Cardiff, Wales, UK	100 gym members (50 crosfitt members and 50 traditional gym members)	Quan	
[101]	Wininger	2002	Instructors' and Classroom Characteristics Associated with Exercise Enjoyment by Females	USA, south eastern part	296 women (M age=21.89 yr., SD=3.52 yr.) were attending not-for-credit aerobics classes	Quan	
[102]	Yin	2001	Setting for exercise and concerns about body appearance of women who exercise	USA, South Texas	74 female fitness center members divided in 2 groups:<br>Women-only Area group (n=36) mean age=28.8 years<br>Co-ed Area group (n=38): mean age = 30.4 years	Quan	

Reference list:

1. Allen JE. An exercise in frustration. Los Angeles Times - Southern California Edition. 2001;:S1–6.

2. Anderson C, Grant RL, Hurley MV. Exercise facilities for neurologically disabled populations - Perceptions from the fitness industry. Disabil Health J. 2017;10:157–62.

3. Andreasson J, Tugetam Å, Bergman P. Keeping Death at Bay through Health Negotiation: Older Adults' Understanding of Health and Life within Gym and Fitness Culture. Act Adapt Aging. 2016;40:200–18.

4. Arbour-Nicitopoulos KP, Ginis KAM. Universal accessibility of "accessible" fitness and recreational facilities for persons with mobility disabilities. Adapt Phys Activ Q. 2011;28:1–15.

5. Asserhøj TL. Danes' fitness habits and use of commercial sports offers [Danskernes fitnessvaner og brug af kommercielle idrætstilbud]. Copenhagen: The Danish Institute for Sports Studies [Idrættens Analyseinstitut]; 2017.

6. Athanasopoulou P, Oikonomou K, Douvis I, Skalkos J. Consumer Behaviour in Fitness Centers: A Typologi of Customers. In: Session 10: Competitive Papers: Sports Marketing. Liverpool; 2011. https://marketing.conference-services.net/programme.asp?conferenceID=2342&action=prog_list&session=15841.

7. Berke EM, Ackermann RT, Lin EH, Diehr PH, Maciejewski ML, Williams B, et al. Distance as a barrier to using a fitness-program benefit for managed Medicare enrollees. Journal of aging and physical activity. 2006;14:313–24.

8. Bethancourt HJ, Rosenberg DE, Beatty T, Arterburn DE. Barriers to and facilitators of physical activity program use among older adults. Clin Med Res. 2014;12:10–20.

9. Brown TC, Volberding J, Baghurst T, Sellers J. Comparing current fitness center members' perceptions of the motivational climate with non-members. Global health promotion. 2017;24:5–13.

10. Brown T c., Volberding J, Baghurst T, Sellers J. Faculty/staff perceptions of a free campus fitness facility. International Journal of Workplace Health Management. 2014;7:156–70.

11. Brownfield P. Setting his own pace in the gym rat race. Los Angeles Times -- Southern California Edition (Front Page). 2002;:S1–6.

12. Calder A, Sole G, Mulligan H. The accessibility of fitness centers for people with disabilities: A systematic review. Disability and Health Journal. 2018;11:525–36.

13. Campos F, Martins F, Simões V, Franco S. Fitness participants perceived quality by age and practiced activity. Journal of Physical Education and Sport. 2017;17:698–704.

14. Caudwell KM, Keatley DA. The Effect of Men's Body Attitudes and Motivation for Gym Attendance. Journal of strength and conditioning research. 2016;30:2550–6.

15. Choitz P, Johnson MP, Berhane Z, Lefever G, Anderson JK, Eiser AR. Urban Fitness Centers: Removing Barriers to Promote Exercise in Underserved Communities. Journal of Health Care for the Poor and Underserved. 2010;21:221–8.

16. Courneya KS, Estabrooks PA, Nigg CR. A Simple Reinforcement Strategy for Increasing Attendance at a Fitness Facility. Health Education & Behavior. 1997;24:708–15.

17. Cyr A, Munroe-Chandler K, Gammage K. Might plight: The social anxiety felt by men in the weightlifting environment. Acsm's Health & Fitness Journal. 2019;23:14–8.

18. Doğan C. Training at the Gym, Training for Life: Creating Better Versions of the Self Through Exercise. Eur J Psychol. 2015;11:442–58.

19. Dolbow DR, Figoni SF. Accommodation of wheelchair-reliant individuals by community fitness facilities. Spinal Cord. 2015;53:515–9.

20. Emeterio ICS, García-Unanue J, Iglesias-Soler E, Felipe JL, Gallardo L. Prediction of abandonment in Spanish fitness centres. European Journal of Sport Science. 2019;19:217–24.

21. Evans MB, Graupensperger S, Benson AJ, Eys M, Hastings B, Gottschall JS. Groupness perceptions and basic need satisfaction: Perceptions of fitness groups and experiences within club environments. Group Dynamics: Theory, Research, and Practice. 2019;:No Pagination Specified-No Pagination Specified.

22. Evans MB, Shanahan E, Leith S, Litvak N, Wilson AE. Living for Today or Tomorrow? Self-Regulation amidst Proximal or Distal Exercise Outcomes. Applied Psychology: Health and Well-Being. 2019;11:304–27.

23. Faulkner G, Dale LP, Lau E. Examining the use of loyalty point incentives to encourage health and fitness centre participation. Preventive Med Reports. 2019;14. doi:10.1016/j.pmedr.2019.100831.

24. Fieril KP, Olsén MF, Glantz A, Larsson M. Experiences of exercise during pregnancy among women who perform regular resistance training: A qualitative study. Phys Ther. 2014;94:1135–43.

25. Fredslund EK, Leppin A. Can the Easter break induce a long-term break of exercise routines? An analysis of Danish gym data using a regression discontinuity design. BMJ Open. 2019;9:e024043.

26. Fullerton A, Macdonald M, Brown A, Ho P-L, Martin J, Tang A, et al. Survey of fitness facilities for individuals post-stroke in the Greater Toronto Area. Applied Physiology, Nutrition, and Metabolism. 2008;33:713–9.

27. Gjestvang C, Stensrud T, Haakstad LAH. Are changes in physical fitness, body composition and weight associated with exercise attendance and dropout among fitness club members? Longitudinal prospective study. BMJ Open. 2019;9:e027987.

28. Gross J, Kroll T, Morris J. Accessibility of fitness centres for people with disabilities in a region in North East Scotland. Public Health. 2013;127:782–4.

29. Harada K, Shibata A, Ishii K, Liao Y, Oka K. Perceived and objectively measured access to strength-training facilities and strength-training behavior. Annals of Behavioral Medicine. 2014;48:120–4.

30. Heinrich KM, Carlisle T, Kehler A, Cosgrove SJ. Mapping Coaches' Views of Participation in CrossFit to the Integrated Theory of Health Behavior Change and Sense of Community: Family & Community Health. 2017;40:24–7.

31. Hosek PP. Self-motivation and exercise adherence in adult women. Dissertation Abstracts International Section A: Humanities and Social Sciences. 1997;58.

32. Hurley SL, Axelson PW. Universal design of fitness equipment criteria to meet the new department of justice accessibility requirements. In: Rehabilitation Engineering and Assistive Technology Society of North Americe (RESNA) Annual conference. Beneficial Designs, Inc.; 2012. p. 4.

33. Hurrell RM. Factors Associated with Regular Exercise. Perceptual and Motor Skills. 1997;84:871–4.

34. Inclusive Fitness Coalition. Facts for Fitness Centers Information Sheet: Making Your Health & Fitness Center a Welcoming Facility. 2015. http://incfit.org/node/733.

35. Jang WY, Choi K. Factors influencing choice when enrolling at a fitness center. Soc Behav Pers. 2018;46:1043–56.

36. Jekauc D, Völkle M, Wagner MO, Mess F, Reiner M, Renner B. Prediction of attendance at fitness center: a comparison between the theory of planned behavior, the social cognitive theory, and the physical activity maintenance theory. Front Psychol. 2015;6. doi:10.3389/fpsyg.2015.00121.

37. Johnson M, Stoelzle H, Finco K, Foss S, Carstens K. ADA Compliance and Accessibility of Fitness Facilities in Western Wisconsin. Topics in Spinal Cord Injury Rehabilitation. 2012;18:340–53.

38. Johnston KR, Goodwin DL, Leo J. Understanding dignity: experiences of impairment in an exercise facility. Adapt Phys Activ Q. 2015;32:106–24.

39. Kailes JI. Using a Fitness Center Does Not Have to be an Exercise in Frustration: Tips for People with Mobility and Visual Disabilities. Published and distributed by Center for Disability Issues and the Health Profession, Western University of Health Sciences; 2008.

40. Kaushal N, Rhodes R. Exercise habit formation in new gym members: a longitudinal study. Journal of Behavioral Medicine. 2015;38:652–63.

41. Kaushal N, Rhodes RE, Meldrum JT, Spence JC. The role of habit in different phases of exercise. British Journal of Health Psychology. 2017;22:429–48.

42. Kirkegaard KL. Part 2: Portray of the active fitness customers: motives for training, satisfaction and self-reported health [Delrapport 2: Portræt af de aktive fitnesskunder: træningsmotiver, tilfredshed og selvvurderet sundhed]. In: Health for millions [Sundhed for millioner]. Copenhagen: The Danish Institute for Sports Studies [Idrættens Analyseinstitut]; 2009.

43. Kirkegaard KL, Østerlund K. Part 5: Fitness culture between sports club and business: active fitness users motives for training, satisfaction and self-reported health [Delrapport 5: Fitnesskultur mellem forening og forretning: aktive fitnessudøveres træningsmotiver, tilfredshed og selvvurderede sundhed]. In: Health for millions [Sundhed for millioner]. Copenhagen: The Danish Institute for Sports Studies [Idrættens Analyseinstitut]; 2010.

44. Klein J. Make a Positive Connection. American Fitness. 2002;20:35.

45. Kruisselbrink LD, Dodge AM, Swanburg SL, MacLeod AL. Influence of Same-Sex and Mixed-Sex Exercise Settings on the Social Physique Anxiety and Exercise Intentions of Males and Females. Journal of Sport & Exercise Psychology. 2004;26:616.

46. Larson HK, McFadden K, McHugh TLF, Berry TR, Rodgers WM. You can't always get what you want: expectations, outcomes, and adherence of new exercisers. Qual Res Sport Exerc Health. 2017;9:389–402.

47. León-Quismondo J, García-Unanue J, Burillo P. Service Perceptions in Fitness Centers: IPA Approach by Gender and Age. Int J Environ Res Public Health. 2020;17.

48. Lockett M. Information Package on AIMFREE Accessibility Instruments Measuring Fitness and Recreation Environments. KT4TT Center, University of Buffalo; 2011.

49. Lopez-Fernandez J, Staniland B, Sanchez I, Iturriaga T, Ayuso M, Horton E, et al. A Weekly Structured Physical Activity Program Enhances Short-term Retention Of Middle-aged Adult Fitness Centre Users: 284 Board #125 May 30 9: 30 AM - 11: 00 AM. Medicine & Science in Sports & Exercise. 2018;50:55.

50. Lübcke A, Martin C, Hellström K. Older Adults' Perceptions of Exercising in a Senior Gym. Act Adapt Aging. 2012;36:131–46.

51. Malek MH, Nalbone DP, Berger DE, Coburn JW. Importance of Health Science Education for Personal Fitness Trainers. Journal of Strength & Conditioning Research. 2002;16:19–24.

52. Manning KM, Chmelo E, Yates BA, Conteh P, Morey M. Adopting a functional fitness approach to membership. Journal on Active Aging. 2019;18:74–9.

53. Martin P, McCann TV. Exercise and older women's wellbeing. Contemporary nurse. 2005;20:169–79.

54. McDonnell AB. Family-friendly locker rooms: as the demographics in your facility change to include more families and older adults, catering to their locker room needs may be to your advantage. Fitness Management. 2002;18:44–7.

55. Melton DI, Dail TK, Katula JA, Mustian KM. The current state of personal training: managers' perspectives. Journal of Strength & Conditioning Research. 2010;24:3173–9.

56. Melton DI, Katula JA, Mustian KM. The Current State of Personal Training: An Industry Perspective of Personal Trainers in a Small Southeast Community: Journal of Strength and Conditioning Research. 2008;22:883–9.

57. Middelkamp J, van Rooijen M, Wolfhagen P, Steenbergen B. The Effects of Two Self-Regulation Interventions to Increase Self-Efficacy and Group Exercise Behavior in Fitness Clubs. Journal of Sports Science & Medicine. 2016;15:358–64.

58. Milner C. Equipping your fitness centre for older adults. Fitness Business Canada. 2005;6:58–9.

59. Mullen SP, Whaley DE. Age, gender, and fitness club membership: Factors related to initial involvement and sustained participation. International Journal of Sport and Exercise Psychology. 2010;8:24–35.

60. Nary D, Froehlich AK, White G. Accessibility of Fitness Facilities for Persons with Physical Disabilities Using Wheelchairs. Topics in Spinal Cord Injury Rehabilitation. 2000;6:87–98.

61. North Carolina Office on Disability and Health, The Center for Universal Design. Removing barriers to Health Clubs and Fitness Facilities - A guide for Accommodating All Members, Including People with Disabilities and Older Adults. The University of North Carolina; 2008. https://fpg.unc.edu/resources/removing-barriers-health-clubs-and-fitness-facilities-guide-accommodating-all-members-incl.

62. Østerlund K, Kirkegaard KL. Part 4 Fitness centers within company sports - Portray of the active fitness users: motives for training, satisfaction and self-reported health [Delrapport 4: Fitnesscentre i firmaidrætten - portræt af de aktive medlemmer: træningsmotiver, tilfredshed og selvvurderet sundhed]. In: Health for millions [Sundhed for millioner]. Copenhagen: The Danish Institute for Sports Studies [Idrættens Analyseinstitut]; 2010.

63. Østerlund K, Kirkegaard KL. Part 3: Fitness in sports clubs/associations - Portray of the active fitness users: motives for training, satisfaction and self-reported health [Delrapport 3 Foreningsfitness - portæt af de aktive medlemmer: træningsmotiver, tilfredshed og selvvurderet sundhed]. In: Health for millions [Sundhed for millioner]. Copenhagen: The Danish Institute for Sports Studies [Idrættens Analyseinstitut]; 2010.

64. Pettigrew S, Burton E, Farrier K, Hill AM, Bainbridge L, Lewin G, et al. A typology of factors influencing seniors' participation in strength training in gyms and fitness centers. J Aging Phys Act. 2018;26:492–8.

65. Rabiee F, Robbins A, Khan M. Gym for Free: The short-term impact of an innovative public health policy on the health and wellbeing of residents in a deprived constituency in Birmingham, UK. Health Education Journal. 2015;74:691–704.

66. Rasmussen RL, Overgaard C, Bøggild H, Thomsen LLH. An explorative evaluation study of the mechanisms underlying a community-based fitness centre in Denmark – Why do residents participate and keep up the healthy activities? Health & Social Care in the Community. 2018;26:898–907.

67. Rauworth A. Designing a fitness facility for all. ACMS's certified news. 2006;16:5–7.

68. Rekieta SK. Exercise relapse prevention: The efficacy of a motivational interview intervention. Dissertation Abstracts International: Section B: The Sciences and Engineering. 2002;63.

69. Richardson EV, Smith B, Papathomas A. Crossing boundaries: The perceived impact of disabled fitness instructors in the gym. Psychology of Sport and Exercise. 2017;29:84–92.

70. Richardson EV, Smith B, Papathomas A. Collective Stories of Exercise: Making Sense of Gym Experiences With Disabled Peers. Adapt Phys Activ Q. 2017;34:276–94.

71. Richardson EV, Smith B, Papathomas A. Disability and the gym: experiences, barriers and facilitators of gym use for individuals with physical disabilities. Disabil Rehabil. 2017;39:1950–7.

72. Riley BB, Rimmer JH, Wang E, Schiller WJ. A conceptual framework for improving the accessibility of fitness and recreation facilities for people with disabilities. J Phys Act Health. 2008;5:158–68.

73. Rimmer JH, Riley B, Wang E, Rauworth A, Jurkowski J. Physical activity participation among persons with disabilities. American Journal of Preventive Medicine. 2004;26:419–25.

74. Rimmer JH, Padalabalanarayanan S, Malone LA, Mehta T. Fitness facilities still lack accessibility for people with disabilities. Disability and Health Journal. 2017;10:214–21.

75. Riseth L, Nøst TH, Nilsen TIL, Steinsbekk A. Long-term members' use of fitness centers: a qualitative study. BMC Sports Science, Medicine and Rehabilitation. 2019;11. doi:10.1186/s13102-019-0114-z.

76. Rodrigues F, Teixeira DS, Cid L, Monteiro D. Have you been exercising lately? Testing the role of past behavior on exercise adherence. Journal of Health Psychology. 2019;:135910531987824.

77. Rodrigues F, Teixeira DS, Cid L, Machado S, Monteiro D. The role of dark-side of motivation and intention to continue in exercise: A self-determination theory approach. Scandinavian Journal of Psychology. 2019;60:585–95.

78. Rodrigues F, Teixeira DS, Neiva HP, Cid L, Monteiro D. The bright and dark sides of motivation as predictors of enjoyment, intention, and exercise persistence. Scandinavian Journal of Medicine & Science in Sports. 2020;30:787–800.

79. Schmidt MLK, Østergren P, Cormie P, Ragle A-M, Sønksen J, Midtgaard J. "Kicked out into the real world": prostate cancer patients' experiences with transitioning from hospital-based supervised exercise to unsupervised exercise in the community. Supportive Care in Cancer. 2019;27:199–208.

80. Schvey NA, Sbrocco T, Bakalar JL, Ress R, Barmine M, Gorlick J, et al. The experience of weight stigma among gym members with overweight and obesity. Stigma and Health. 2017;2:292–306.

81. Schwetschenau HM, O'Brien WH, Cunningham CJL, Jex SM. Barriers to physical activity in an on-site corporate fitness center. J Occup Health Psychol. 2008;13:371–80.

82. Souza BJ, Ebbeck V. Perspectives on Increasing Positive Attitudes Toward Larger Members in Fitness Centers. Journal of Applied Sport Psychology. 2018;30:96–118.

83. Sperandei S, Vieira MC, Reis AC. Adherence to physical activity in an unsupervised setting: Explanatory variables for high attrition rates among fitness center members. J Sci Med Sport. 2016;19:916–20.

84. Springer JB, Lamborn SD, Pollard DM. Maintaining physical activity over time: The importance of basic psychological need satisfaction in developing the

physically active self. [References]. American Journal of Health Promotion. 2013;27:284–93.

85. Stein J. Bodywork. A swing toward families: gyms are taking a fresh look at classes for kids and parents to encourage old and young to plan their exercise time together. Los Angeles Times -- Southern California Edition (Front Page). 2003;:F7–F7.

86. Stein J. Bodywork. Bracing for the attack of the gym "newbies." Los Angeles Times -- Southern California Edition (Front Page). 2003;:F1–5.

87. Stenson J. Workout partners: health clubs and videos are incorporating kids into routines so time-strapped parents can Strollercize, lift weights or do yoga with children in tow. Los Angeles Times -- Southern California Edition (Front Page). 2005;:F9–F9.

88. Stewart B, Smith ACT. The significance of critical incidents in explaining gym use amongst adult populations. Qual Res Sport Exerc Health. 2014;6:45–61.

89. Strelsand B. No Six-Packs Here, Please. U.S. News & World Report. 2007;142:66–7.

90. Swoyer JO. Equality of fitenss centers: are all fitness centers created equal? The Exceptional Parent. 2008.

91. Thomson D, McAdoo K. An exploration into the development of motivation to exercise in a group of male UK regular gym users. International Journal of Sport and Exercise Psychology. 2016;14:414–29.

92. Tolle H, Rapacz A, Weintraub B, Shogren C, Harkema SJ, Gibson JL. Establishing the NeuroRecovery Network Community Fitness and Wellness facilities: multi-site fitness facilities provide activity-based interventions and assessments for evidence-based functional gains in neurologic disorders. Disability and Rehabilitation. 2018;40:3086–93.

93. Ulseth A-LB. New Opportunities - Complex Motivations: Gender Differences in Motivation for Physical Activity in the Context of Sports Clubs and Fitness Centers. International Journal of Applied Sports Sciences. 2008;20:44–66.

94. Unger JB, Johnson CA. Social relationships and physical activity in health club members. Am J Health Promot. 1995;9:340–3.

95. United states access board. Accessible sports facilities: a summary of accessibility guidelines for recreation facilities. 2003. https://www.access-board.gov/guidelines-and-standards/recreation-facilities/guides/sports-facilities.

96. van der Swaluw K, Lambooij MS, Mathijssen JJP, Schipper M, Zeelenberg M, Berkhout S, et al. Commitment Lotteries Promote Physical Activity Among Overweight Adults—A Cluster Randomized Trial. Annals of Behavioral Medicine. 2018;52:342–51.

97. van der Swaluw K, Lambooij MS, Mathijssen JJP, Schipper M, Zeelenberg M, Berkhout S, et al. Physical activity after commitment lotteries: examining long-term results in a cluster randomized trial. Journal of Behavioral Medicine. 2018;41:483–93.

98. Vlachopoulos SP, Neikou E. A prospective study of the relationships of autonomy, competence, and relatedness with exercise attendance, adherence, and dropout. J Sports Med Phys Fitness. 2007;47:475–82.

99. Wayment HA, McDonald RL. Sharing a personal trainer: Personal and social benefits of individualized, small-group training. Journal of Strength & Conditioning Research. 2017;31:3137–45.

100. Whiteman-Sandland J, Hawkins J, Clayton D. The role of social capital and community belongingness for exercise adherence: An exploratory study of the CrossFit gym model. Journal of Health Psychology. 2018;23:1545–56.

101. Wininger SR. Instructors' and Classroom Characteristics Associated with Exercise Enjoyment by Females. Perceptual & Motor Skills. 2002;94:395–8.

102. Yin Z. Setting for exercise and concerns about body appearance of women who exercise. Perceptual and motor skills. 2001;93:851–5.
